# Supplementary material for: Multiscale Modelling Tool: Mathematical modelling of collective behaviour without the maths
Source: PLoS One. 2019 Sep 30;14(9):e0222906. doi: 10.1371/journal.pone.0222906 (PMC6768458; doi:10.1371/journal.pone.0222906)
Supplement: S1 Text — van Kampen expansion and other analyses of the stop-signal model (Eq 1). (PDF) [file pone.0222906.s001.pdf]

S1 TEXT FOR 'MULTISCALE MODELLING TOOL: MATHEMATICAL MODELLING OF COLLECTIVE BEHAVIOUR WITHOUT THE MATHS'

JAMES A. R. MARSHALL, ANDREAGIOVANNI REINA, THOMAS BOSE

Contents

1.	Master equation for stop-signal model of Seeley *et al.*	1
2.	van Kampen expansion for stop-signal model of Seeley *et al.*	1
3.	Fokker-Planck equation for stop-signal model of Seeley *et al.*	9
References		12

1. Master equation for stop-signal model of Seeley *et al.*

$$\frac{\partial}{\partial t}P(A,B,U,t) :=$$
$$\frac{1}{-\frac{\Delta}{2}+\mu}(\mathrm{E_{op}}(B,1)\,\mathrm{E_{op}}(U,-1)-1)BP(A,B,U,t)+$$
$$s(\mathrm{E_{op}}(A,0)\,\mathrm{E_{op}}(B,1)\,\mathrm{E_{op}}(U,-1)-1)\frac{AB}{V}P(A,B,U,t)+\frac{\Delta}{2}+$$
$$\mu(\mathrm{E_{op}}(A,-1)\,\mathrm{E_{op}}(U,1)-1)\frac{AU}{V}P(A,B,U,t)+\frac{\Delta}{2}+\mu(\mathrm{E_{op}}(A,-1)\,\mathrm{E_{op}}(U,1)-$$
$$1)UP(A,B,U,t)+-\frac{\Delta}{2}+\mu(\mathrm{E_{op}}(B,-1)\,\mathrm{E_{op}}(U,1)-1)\frac{BU}{V}P(A,B,U,t)+$$
$$s(\mathrm{E_{op}}(A,1)\,\mathrm{E_{op}}(B,0)\,\mathrm{E_{op}}(U,-1)-1)\frac{AB}{V}P(A,B,U,t)+$$
$$\frac{1}{\frac{\Delta}{2}+\mu}(\mathrm{E_{op}}(A,1)\,\mathrm{E_{op}}(U,-1)-1)AP(A,B,U,t)+-\frac{\Delta}{2}+$$
$$\mu(\mathrm{E_{op}}(B,-1)\,\mathrm{E_{op}}(U,1)-1)UP(A,B,U,t)$$

using substitution $U := -A-B+N$.

2. van Kampen expansion for stop-signal model of Seeley *et al.*

$$-\sqrt{V}\frac{d}{dt}\Phi_A\frac{\partial}{\partial\eta_A}P(\eta_A,\eta_B,\eta_U,t)-\sqrt{V}\frac{d}{dt}\Phi_B\frac{\partial}{\partial\eta_B}P(\eta_A,\eta_B,\eta_U,t)-$$
$$\sqrt{V}\frac{d}{dt}\Phi_U\frac{\partial}{\partial\eta_U}P(\eta_A,\eta_B,\eta_U,t)+\frac{\partial}{\partial t}P(\eta_A,\eta_B,\eta_U,t):=$$
$$-\frac{\Delta\Phi_A}{2}\Phi_U\sqrt{V}\frac{\partial}{\partial\eta_A}P(\eta_A,\eta_B,\eta_U,t)+\frac{\Delta\Phi_A}{2}\Phi_U\sqrt{V}\frac{\partial}{\partial\eta_U}P(\eta_A,\eta_B,\eta_U,t)+$$
$$\frac{\Delta\Phi_A}{4}\Phi_U\frac{\partial^2}{\partial\eta_A^2}P(\eta_A,\eta_B,\eta_U,t)-\frac{\Delta\Phi_A}{2}\Phi_U\frac{\partial^2}{\partial\eta_A\partial\eta_U}P(\eta_A,\eta_B,\eta_U,t)+$$
$$\frac{\Delta\Phi_A}{4}\Phi_U\frac{\partial^2}{\partial\eta_U^2}P(\eta_A,\eta_B,\eta_U,t)+\frac{\Delta\Phi_A\Phi_U}{8V}\frac{\partial^4}{\partial\eta_A^2\partial\eta_U^2}P(\eta_A,\eta_B,\eta_U,t)+$$
$$\frac{\Delta\Phi_A\Phi_U}{4\sqrt{V}}\frac{\partial^3}{\partial\eta_A^2\partial\eta_U}P(\eta_A,\eta_B,\eta_U,t)-\frac{\Delta\Phi_A\Phi_U}{4\sqrt{V}}\frac{\partial^3}{\partial\eta_A\partial\eta_U^2}P(\eta_A,\eta_B,\eta_U,t)-$$

$$\frac{\Delta\Phi_A}{2}\eta_U\frac{\partial}{\partial\eta_A}P(\eta_A,\eta_B,\eta_U,t)+\frac{\Delta\Phi_A}{2}\eta_U\frac{\partial}{\partial\eta_U}P(\eta_A,\eta_B,\eta_U,t)+$$
$$\frac{\Delta\Phi_A}{2}P(\eta_A,\eta_B,\eta_U,t)+\frac{\Delta\Phi_A\eta_U}{4\overline{V}}\frac{\partial^3}{\partial\eta_A^2\partial\eta_U}P(\eta_A,\eta_B,\eta_U,t)-$$
$$\frac{\Delta\Phi_A\eta_U}{4\overline{V}}\frac{\partial^3}{\partial\eta_A\partial\eta_U^2}P(\eta_A,\eta_B,\eta_U,t)+\frac{\Delta\Phi_A}{4\overline{V}}\frac{\partial^2}{\partial\eta_A^2}P(\eta_A,\eta_B,\eta_U,t)-$$
$$\frac{\Delta\Phi_A}{2\overline{V}}\frac{\partial^2}{\partial\eta_A\partial\eta_U}P(\eta_A,\eta_B,\eta_U,t)+\frac{\Delta\Phi_A\eta_U}{4\sqrt{\overline{V}}}\frac{\partial^2}{\partial\eta_A^2}P(\eta_A,\eta_B,\eta_U,t)-$$
$$\frac{\Delta\Phi_A\eta_U}{2\sqrt{\overline{V}}}\frac{\partial^2}{\partial\eta_A\partial\eta_U}P(\eta_A,\eta_B,\eta_U,t)+\frac{\Delta\Phi_A\eta_U}{4\sqrt{\overline{V}}}\frac{\partial^2}{\partial\eta_U^2}P(\eta_A,\eta_B,\eta_U,t)-$$
$$\frac{\Delta\Phi_A}{2\sqrt{\overline{V}}}\frac{\partial}{\partial\eta_A}P(\eta_A,\eta_B,\eta_U,t)+\frac{\Delta\Phi_A}{2\sqrt{\overline{V}}}\frac{\partial}{\partial\eta_U}P(\eta_A,\eta_B,\eta_U,t)+$$
$$\frac{\Delta\Phi_A\eta_U}{8\overline{V}^{\frac{3}{2}}}\frac{\partial^4}{\partial\eta_A^2\partial\eta_U^2}P(\eta_A,\eta_B,\eta_U,t)+\frac{\Delta\Phi_A}{4\overline{V}^{\frac{3}{2}}}\frac{\partial^3}{\partial\eta_A^2\partial\eta_U}P(\eta_A,\eta_B,\eta_U,t)+$$
$$\frac{\Delta\Phi_B}{2}\Phi_U\sqrt{\overline{V}}\frac{\partial}{\partial\eta_B}P(\eta_A,\eta_B,\eta_U,t)-\frac{\Delta\Phi_B}{2}\Phi_U\sqrt{\overline{V}}\frac{\partial}{\partial\eta_U}P(\eta_A,\eta_B,\eta_U,t)-$$
$$\frac{\Delta\Phi_B}{4}\Phi_U\frac{\partial^2}{\partial\eta_B^2}P(\eta_A,\eta_B,\eta_U,t)+\frac{\Delta\Phi_B}{2}\Phi_U\frac{\partial^2}{\partial\eta_B\partial\eta_U}P(\eta_A,\eta_B,\eta_U,t)-$$
$$\frac{\Delta\Phi_B}{4}\Phi_U\frac{\partial^2}{\partial\eta_U^2}P(\eta_A,\eta_B,\eta_U,t)-\frac{\Delta\Phi_B\Phi_U}{8\overline{V}}\frac{\partial^4}{\partial\eta_B^2\partial\eta_U^2}P(\eta_A,\eta_B,\eta_U,t)-$$
$$\frac{\Delta\Phi_B\Phi_U}{4\sqrt{\overline{V}}}\frac{\partial^3}{\partial\eta_B^2\partial\eta_U}P(\eta_A,\eta_B,\eta_U,t)+\frac{\Delta\Phi_B\Phi_U}{4\sqrt{\overline{V}}}\frac{\partial^3}{\partial\eta_B\partial\eta_U^2}P(\eta_A,\eta_B,\eta_U,t)+$$
$$\frac{\Delta\Phi_B}{2}\eta_U\frac{\partial}{\partial\eta_B}P(\eta_A,\eta_B,\eta_U,t)-\frac{\Delta\Phi_B}{2}\eta_U\frac{\partial}{\partial\eta_U}P(\eta_A,\eta_B,\eta_U,t)-$$
$$\frac{\Delta\Phi_B}{2}P(\eta_A,\eta_B,\eta_U,t)-\frac{\Delta\Phi_B\eta_U}{4\overline{V}}\frac{\partial^3}{\partial\eta_B^2\partial\eta_U}P(\eta_A,\eta_B,\eta_U,t)+$$
$$\frac{\Delta\Phi_B\eta_U}{4\overline{V}}\frac{\partial^3}{\partial\eta_B\partial\eta_U^2}P(\eta_A,\eta_B,\eta_U,t)-\frac{\Delta\Phi_B}{4\overline{V}}\frac{\partial^2}{\partial\eta_B^2}P(\eta_A,\eta_B,\eta_U,t)+$$
$$\frac{\Delta\Phi_B}{2\overline{V}}\frac{\partial^2}{\partial\eta_B\partial\eta_U}P(\eta_A,\eta_B,\eta_U,t)-\frac{\Delta\Phi_B\eta_U}{4\sqrt{\overline{V}}}\frac{\partial^2}{\partial\eta_B^2}P(\eta_A,\eta_B,\eta_U,t)+$$
$$\frac{\Delta\Phi_B\eta_U}{2\sqrt{\overline{V}}}\frac{\partial^2}{\partial\eta_B\partial\eta_U}P(\eta_A,\eta_B,\eta_U,t)-\frac{\Delta\Phi_B\eta_U}{4\sqrt{\overline{V}}}\frac{\partial^2}{\partial\eta_U^2}P(\eta_A,\eta_B,\eta_U,t)+$$
$$\frac{\Delta\Phi_B}{2\sqrt{\overline{V}}}\frac{\partial}{\partial\eta_B}P(\eta_A,\eta_B,\eta_U,t)-\frac{\Delta\Phi_B}{2\sqrt{\overline{V}}}\frac{\partial}{\partial\eta_U}P(\eta_A,\eta_B,\eta_U,t)-$$
$$\frac{\Delta\Phi_B\eta_U}{8\overline{V}^{\frac{3}{2}}}\frac{\partial^4}{\partial\eta_B^2\partial\eta_U^2}P(\eta_A,\eta_B,\eta_U,t)-\frac{\Delta\Phi_B}{4\overline{V}^{\frac{3}{2}}}\frac{\partial^3}{\partial\eta_B^2\partial\eta_U}P(\eta_A,\eta_B,\eta_U,t)-$$
$$\frac{\Delta\Phi_U}{2}\sqrt{\overline{V}}\frac{\partial}{\partial\eta_A}P(\eta_A,\eta_B,\eta_U,t)+\frac{\Delta\Phi_U}{2}\sqrt{\overline{V}}\frac{\partial}{\partial\eta_B}P(\eta_A,\eta_B,\eta_U,t)-$$
$$\frac{\Delta\Phi_U}{2}\eta_A\frac{\partial}{\partial\eta_A}P(\eta_A,\eta_B,\eta_U,t)+\frac{\Delta\Phi_U}{2}\eta_A\frac{\partial}{\partial\eta_U}P(\eta_A,\eta_B,\eta_U,t)+$$
$$\frac{\Delta\Phi_U}{2}\eta_B\frac{\partial}{\partial\eta_B}P(\eta_A,\eta_B,\eta_U,t)-\frac{\Delta\Phi_U}{2}\eta_B\frac{\partial}{\partial\eta_U}P(\eta_A,\eta_B,\eta_U,t)+$$
$$\frac{\Delta\Phi_U}{4}\frac{\partial^2}{\partial\eta_A^2}P(\eta_A,\eta_B,\eta_U,t)-\frac{\Delta\Phi_U}{2}\frac{\partial^2}{\partial\eta_A\partial\eta_U}P(\eta_A,\eta_B,\eta_U,t)-$$
$$\frac{\Delta\Phi_U}{4}\frac{\partial^2}{\partial\eta_B^2}P(\eta_A,\eta_B,\eta_U,t)+\frac{\Delta\Phi_U}{2}\frac{\partial^2}{\partial\eta_B\partial\eta_U}P(\eta_A,\eta_B,\eta_U,t)+$$
$$\frac{\Delta\Phi_U\eta_A}{4\overline{V}}\frac{\partial^3}{\partial\eta_A^2\partial\eta_U}P(\eta_A,\eta_B,\eta_U,t)-\frac{\Delta\Phi_U\eta_A}{4\overline{V}}\frac{\partial^3}{\partial\eta_A\partial\eta_U^2}P(\eta_A,\eta_B,\eta_U,t)-$$
$$\frac{\Delta\Phi_U\eta_B}{4\overline{V}}\frac{\partial^3}{\partial\eta_B^2\partial\eta_U}P(\eta_A,\eta_B,\eta_U,t)+\frac{\Delta\Phi_U\eta_B}{4\overline{V}}\frac{\partial^3}{\partial\eta_B\partial\eta_U^2}P(\eta_A,\eta_B,\eta_U,t)+$$
$$\frac{\Delta\Phi_U}{2\overline{V}}\frac{\partial^2}{\partial\eta_A\partial\eta_U}P(\eta_A,\eta_B,\eta_U,t)-\frac{\Delta\Phi_U}{2\overline{V}}\frac{\partial^2}{\partial\eta_B\partial\eta_U}P(\eta_A,\eta_B,\eta_U,t)+$$
$$\frac{\Delta\Phi_U}{8\overline{V}}\frac{\partial^4}{\partial\eta_A^2\partial\eta_U^2}P(\eta_A,\eta_B,\eta_U,t)-\frac{\Delta\Phi_U}{8\overline{V}}\frac{\partial^4}{\partial\eta_B^2\partial\eta_U^2}P(\eta_A,\eta_B,\eta_U,t)+$$
$$\frac{\Delta\Phi_U\eta_A}{4\sqrt{\overline{V}}}\frac{\partial^2}{\partial\eta_A^2}P(\eta_A,\eta_B,\eta_U,t)-\frac{\Delta\Phi_U\eta_A}{2\sqrt{\overline{V}}}\frac{\partial^2}{\partial\eta_A\partial\eta_U}P(\eta_A,\eta_B,\eta_U,t)+$$

$$\frac{\Delta\Phi_U\eta_A}{4\sqrt{V}}\frac{\partial^2}{\partial\eta_U^2}P(\eta_A,\eta_B,\eta_U,t)-\frac{\Delta\Phi_U\eta_B}{4\sqrt{V}}\frac{\partial^2}{\partial\eta_B^2}P(\eta_A,\eta_B,\eta_U,t)+$$
$$\frac{\Delta\Phi_U\eta_B}{2\sqrt{V}}\frac{\partial^2}{\partial\eta_B\partial\eta_U}P(\eta_A,\eta_B,\eta_U,t)-\frac{\Delta\Phi_U\eta_B}{4\sqrt{V}}\frac{\partial^2}{\partial\eta_U^2}P(\eta_A,\eta_B,\eta_U,t)+$$
$$\frac{\Delta\Phi_U}{2\sqrt{V}}\frac{\partial}{\partial\eta_A}P(\eta_A,\eta_B,\eta_U,t)-\frac{\Delta\Phi_U}{2\sqrt{V}}\frac{\partial}{\partial\eta_B}P(\eta_A,\eta_B,\eta_U,t)+$$
$$\frac{\Delta\Phi_U}{4\sqrt{V}}\frac{\partial^3}{\partial\eta_A^2\partial\eta_U}P(\eta_A,\eta_B,\eta_U,t)-\frac{\Delta\Phi_U}{4\sqrt{V}}\frac{\partial^3}{\partial\eta_A\partial\eta_U^2}P(\eta_A,\eta_B,\eta_U,t)-$$
$$\frac{\Delta\Phi_U}{4\sqrt{V}}\frac{\partial^3}{\partial\eta_B^2\partial\eta_U}P(\eta_A,\eta_B,\eta_U,t)+\frac{\Delta\Phi_U}{4\sqrt{V}}\frac{\partial^3}{\partial\eta_B\partial\eta_U^2}P(\eta_A,\eta_B,\eta_U,t)+$$
$$\frac{\Delta\Phi_U\eta_A}{8V^{\frac{3}{2}}}\frac{\partial^4}{\partial\eta_A^2\partial\eta_U^2}P(\eta_A,\eta_B,\eta_U,t)-\frac{\Delta\Phi_U\eta_B}{8V^{\frac{3}{2}}}\frac{\partial^4}{\partial\eta_B^2\partial\eta_U^2}P(\eta_A,\eta_B,\eta_U,t)+$$
$$\frac{\Delta\Phi_U}{4V^{\frac{3}{2}}}\frac{\partial^3}{\partial\eta_A\partial\eta_U^2}P(\eta_A,\eta_B,\eta_U,t)-\frac{\Delta\Phi_U}{4V^{\frac{3}{2}}}\frac{\partial^3}{\partial\eta_B\partial\eta_U^2}P(\eta_A,\eta_B,\eta_U,t)-$$
$$\frac{\Delta\eta_U}{2}\frac{\partial}{\partial\eta_A}P(\eta_A,\eta_B,\eta_U,t)+\frac{\Delta\eta_U}{2}\frac{\partial}{\partial\eta_B}P(\eta_A,\eta_B,\eta_U,t)+$$
$$\frac{\Delta\eta_A\eta_U}{4V}\frac{\partial^2}{\partial\eta_A^2}P(\eta_A,\eta_B,\eta_U,t)-\frac{\Delta\eta_A\eta_U}{2V}\frac{\partial^2}{\partial\eta_A\partial\eta_U}P(\eta_A,\eta_B,\eta_U,t)+$$
$$\frac{\Delta\eta_A\eta_U}{4V}\frac{\partial^2}{\partial\eta_U^2}P(\eta_A,\eta_B,\eta_U,t)-\frac{\Delta\eta_A}{2V}\frac{\partial}{\partial\eta_A}P(\eta_A,\eta_B,\eta_U,t)+$$
$$\frac{\Delta\eta_A}{2V}\frac{\partial}{\partial\eta_U}P(\eta_A,\eta_B,\eta_U,t)-\frac{\Delta\eta_B\eta_U}{4V}\frac{\partial^2}{\partial\eta_B^2}P(\eta_A,\eta_B,\eta_U,t)+$$
$$\frac{\Delta\eta_B\eta_U}{2V}\frac{\partial^2}{\partial\eta_B\partial\eta_U}P(\eta_A,\eta_B,\eta_U,t)-\frac{\Delta\eta_B\eta_U}{4V}\frac{\partial^2}{\partial\eta_U^2}P(\eta_A,\eta_B,\eta_U,t)+$$
$$\frac{\Delta\eta_B}{2V}\frac{\partial}{\partial\eta_B}P(\eta_A,\eta_B,\eta_U,t)-\frac{\Delta\eta_B}{2V}\frac{\partial}{\partial\eta_U}P(\eta_A,\eta_B,\eta_U,t)+$$
$$\frac{\Delta\eta_U}{2V}\frac{\partial}{\partial\eta_A}P(\eta_A,\eta_B,\eta_U,t)-\frac{\Delta\eta_U}{2V}\frac{\partial}{\partial\eta_B}P(\eta_A,\eta_B,\eta_U,t)+$$
$$\frac{\Delta\eta_U}{4V}\frac{\partial^3}{\partial\eta_A^2\partial\eta_U}P(\eta_A,\eta_B,\eta_U,t)-\frac{\Delta\eta_U}{4V}\frac{\partial^3}{\partial\eta_A\partial\eta_U^2}P(\eta_A,\eta_B,\eta_U,t)-$$
$$\frac{\Delta\eta_U}{4V}\frac{\partial^3}{\partial\eta_B^2\partial\eta_U}P(\eta_A,\eta_B,\eta_U,t)+\frac{\Delta\eta_U}{4V}\frac{\partial^3}{\partial\eta_B\partial\eta_U^2}P(\eta_A,\eta_B,\eta_U,t)+$$
$$\frac{\Delta}{4V}\frac{\partial^2}{\partial\eta_A^2}P(\eta_A,\eta_B,\eta_U,t)-\frac{\Delta}{2V}\frac{\partial^2}{\partial\eta_A\partial\eta_U}P(\eta_A,\eta_B,\eta_U,t)-$$
$$\frac{\Delta}{4V}\frac{\partial^2}{\partial\eta_B^2}P(\eta_A,\eta_B,\eta_U,t)+\frac{\Delta}{2V}\frac{\partial^2}{\partial\eta_B\partial\eta_U}P(\eta_A,\eta_B,\eta_U,t)+$$
$$\frac{\Delta\eta_A\eta_U}{8V^2}\frac{\partial^4}{\partial\eta_A^2\partial\eta_U^2}P(\eta_A,\eta_B,\eta_U,t)+\frac{\Delta\eta_A}{4V^2}\frac{\partial^3}{\partial\eta_A^2\partial\eta_U}P(\eta_A,\eta_B,\eta_U,t)-$$
$$\frac{\Delta\eta_B\eta_U}{8V^2}\frac{\partial^4}{\partial\eta_B^2\partial\eta_U^2}P(\eta_A,\eta_B,\eta_U,t)-\frac{\Delta\eta_B}{4V^2}\frac{\partial^3}{\partial\eta_B^2\partial\eta_U}P(\eta_A,\eta_B,\eta_U,t)+$$
$$\frac{\Delta\eta_U}{4V^2}\frac{\partial^3}{\partial\eta_A\partial\eta_U^2}P(\eta_A,\eta_B,\eta_U,t)-\frac{\Delta\eta_U}{4V^2}\frac{\partial^3}{\partial\eta_B\partial\eta_U^2}P(\eta_A,\eta_B,\eta_U,t)+$$
$$\frac{\Delta}{2V^2}\frac{\partial^2}{\partial\eta_A\partial\eta_U}P(\eta_A,\eta_B,\eta_U,t)-\frac{\Delta}{2V^2}\frac{\partial^2}{\partial\eta_B\partial\eta_U}P(\eta_A,\eta_B,\eta_U,t)-$$
$$\frac{\Delta\eta_A\eta_U}{2\sqrt{V}}\frac{\partial}{\partial\eta_A}P(\eta_A,\eta_B,\eta_U,t)+\frac{\Delta\eta_A\eta_U}{2\sqrt{V}}\frac{\partial}{\partial\eta_U}P(\eta_A,\eta_B,\eta_U,t)+$$
$$\frac{\Delta\eta_A}{2\sqrt{V}}P(\eta_A,\eta_B,\eta_U,t)+\frac{\Delta\eta_B\eta_U}{2\sqrt{V}}\frac{\partial}{\partial\eta_B}P(\eta_A,\eta_B,\eta_U,t)-$$
$$\frac{\Delta\eta_B\eta_U}{2\sqrt{V}}\frac{\partial}{\partial\eta_U}P(\eta_A,\eta_B,\eta_U,t)-\frac{\Delta\eta_B}{2\sqrt{V}}P(\eta_A,\eta_B,\eta_U,t)+$$
$$\frac{\Delta\eta_U}{4\sqrt{V}}\frac{\partial^2}{\partial\eta_A^2}P(\eta_A,\eta_B,\eta_U,t)-\frac{\Delta\eta_U}{2\sqrt{V}}\frac{\partial^2}{\partial\eta_A\partial\eta_U}P(\eta_A,\eta_B,\eta_U,t)-$$
$$\frac{\Delta\eta_U}{4\sqrt{V}}\frac{\partial^2}{\partial\eta_B^2}P(\eta_A,\eta_B,\eta_U,t)+\frac{\Delta\eta_U}{2\sqrt{V}}\frac{\partial^2}{\partial\eta_B\partial\eta_U}P(\eta_A,\eta_B,\eta_U,t)-$$
$$\frac{\Delta}{2\sqrt{V}}\frac{\partial}{\partial\eta_A}P(\eta_A,\eta_B,\eta_U,t)+\frac{\Delta}{2\sqrt{V}}\frac{\partial}{\partial\eta_B}P(\eta_A,\eta_B,\eta_U,t)+$$

$$\frac{\Delta\eta_A\eta_U}{4\overline{V}^{\frac{3}{2}}}\frac{\partial^3}{\partial\eta_A^2\partial\eta_U}P(\eta_A,\eta_B,\eta_U,t)-\frac{\Delta\eta_A\eta_U}{4\overline{V}^{\frac{3}{2}}}\frac{\partial^3}{\partial\eta_A\partial\eta_U^2}P(\eta_A,\eta_B,\eta_U,t)+$$
$$\frac{\Delta\eta_A}{4\overline{V}^{\frac{3}{2}}}\frac{\partial^2}{\partial\eta_A^2}P(\eta_A,\eta_B,\eta_U,t)-\frac{\Delta\eta_A}{2\overline{V}^{\frac{3}{2}}}\frac{\partial^2}{\partial\eta_A\partial\eta_U}P(\eta_A,\eta_B,\eta_U,t)-$$
$$\frac{\Delta\eta_B\eta_U}{4\overline{V}^{\frac{3}{2}}}\frac{\partial^3}{\partial\eta_B^2\partial\eta_U}P(\eta_A,\eta_B,\eta_U,t)+\frac{\Delta\eta_B\eta_U}{4\overline{V}^{\frac{3}{2}}}\frac{\partial^3}{\partial\eta_B\partial\eta_U^2}P(\eta_A,\eta_B,\eta_U,t)-$$
$$\frac{\Delta\eta_B}{4\overline{V}^{\frac{3}{2}}}\frac{\partial^2}{\partial\eta_B^2}P(\eta_A,\eta_B,\eta_U,t)+\frac{\Delta\eta_B}{2\overline{V}^{\frac{3}{2}}}\frac{\partial^2}{\partial\eta_B\partial\eta_U}P(\eta_A,\eta_B,\eta_U,t)+$$
$$\frac{\Delta\eta_U}{2\overline{V}^{\frac{3}{2}}}\frac{\partial^2}{\partial\eta_A\partial\eta_U}P(\eta_A,\eta_B,\eta_U,t)-\frac{\Delta\eta_U}{2\overline{V}^{\frac{3}{2}}}\frac{\partial^2}{\partial\eta_B\partial\eta_U}P(\eta_A,\eta_B,\eta_U,t)+$$
$$\frac{\Delta\eta_U}{8\overline{V}^{\frac{3}{2}}}\frac{\partial^4}{\partial\eta_A^2\partial\eta_U^2}P(\eta_A,\eta_B,\eta_U,t)-\frac{\Delta\eta_U}{8\overline{V}^{\frac{3}{2}}}\frac{\partial^4}{\partial\eta_B^2\partial\eta_U^2}P(\eta_A,\eta_B,\eta_U,t)+$$
$$\frac{\Delta}{2\overline{V}^{\frac{3}{2}}}\frac{\partial}{\partial\eta_A}P(\eta_A,\eta_B,\eta_U,t)-\frac{\Delta}{2\overline{V}^{\frac{3}{2}}}\frac{\partial}{\partial\eta_B}P(\eta_A,\eta_B,\eta_U,t)+$$
$$\frac{\Delta}{4\overline{V}^{\frac{3}{2}}}\frac{\partial^3}{\partial\eta_A^2\partial\eta_U}P(\eta_A,\eta_B,\eta_U,t)-\frac{\Delta}{4\overline{V}^{\frac{3}{2}}}\frac{\partial^3}{\partial\eta_B^2\partial\eta_U}P(\eta_A,\eta_B,\eta_U,t)+$$
$$\Phi_A\Phi_B\sqrt{\overline{V}}s\frac{\partial}{\partial\eta_A}P(\eta_A,\eta_B,\eta_U,t)+\Phi_A\Phi_B\sqrt{\overline{V}}s\frac{\partial}{\partial\eta_B}P(\eta_A,\eta_B,\eta_U,t)-$$
$$2\Phi_A\Phi_B\sqrt{\overline{V}}s\frac{\partial}{\partial\eta_U}P(\eta_A,\eta_B,\eta_U,t)+\frac{\Phi_A\Phi_B}{2}s\frac{\partial^2}{\partial\eta_A^2}P(\eta_A,\eta_B,\eta_U,t)-$$
$$\Phi_A\Phi_Bs\frac{\partial^2}{\partial\eta_A\partial\eta_U}P(\eta_A,\eta_B,\eta_U,t)+\frac{\Phi_A\Phi_B}{2}s\frac{\partial^2}{\partial\eta_B^2}P(\eta_A,\eta_B,\eta_U,t)-$$
$$\Phi_A\Phi_Bs\frac{\partial^2}{\partial\eta_B\partial\eta_U}P(\eta_A,\eta_B,\eta_U,t)+\Phi_A\Phi_Bs\frac{\partial^2}{\partial\eta_U^2}P(\eta_A,\eta_B,\eta_U,t)+$$
$$\frac{\Phi_A\Phi_Bs}{4\overline{V}}\frac{\partial^4}{\partial\eta_A^2\partial\eta_U^2}P(\eta_A,\eta_B,\eta_U,t)+\frac{\Phi_A\Phi_Bs}{4\overline{V}}\frac{\partial^4}{\partial\eta_B^2\partial\eta_U^2}P(\eta_A,\eta_B,\eta_U,t)-$$
$$\frac{\Phi_A\Phi_Bs}{2\sqrt{\overline{V}}}\frac{\partial^3}{\partial\eta_A^2\partial\eta_U}P(\eta_A,\eta_B,\eta_U,t)+\frac{\Phi_A\Phi_Bs}{2\sqrt{\overline{V}}}\frac{\partial^3}{\partial\eta_A\partial\eta_U^2}P(\eta_A,\eta_B,\eta_U,t)-$$
$$\frac{\Phi_A\Phi_Bs}{2\sqrt{\overline{V}}}\frac{\partial^3}{\partial\eta_B^2\partial\eta_U}P(\eta_A,\eta_B,\eta_U,t)+\frac{\Phi_A\Phi_Bs}{2\sqrt{\overline{V}}}\frac{\partial^3}{\partial\eta_B\partial\eta_U^2}P(\eta_A,\eta_B,\eta_U,t)-$$
$$\Phi_A\Phi_U\sqrt{\overline{V}}\mu\frac{\partial}{\partial\eta_A}P(\eta_A,\eta_B,\eta_U,t)+\Phi_A\Phi_U\sqrt{\overline{V}}\mu\frac{\partial}{\partial\eta_U}P(\eta_A,\eta_B,\eta_U,t)+$$
$$\frac{\Phi_A\Phi_U}{2}\mu\frac{\partial^2}{\partial\eta_A^2}P(\eta_A,\eta_B,\eta_U,t)-\Phi_A\Phi_U\mu\frac{\partial^2}{\partial\eta_A\partial\eta_U}P(\eta_A,\eta_B,\eta_U,t)+$$
$$\frac{\Phi_A\Phi_U}{2}\mu\frac{\partial^2}{\partial\eta_U^2}P(\eta_A,\eta_B,\eta_U,t)+\frac{\Phi_A\Phi_U\mu}{4\overline{V}}\frac{\partial^4}{\partial\eta_A^2\partial\eta_U^2}P(\eta_A,\eta_B,\eta_U,t)+$$
$$\frac{\Phi_A\Phi_U\mu}{2\sqrt{\overline{V}}}\frac{\partial^3}{\partial\eta_A^2\partial\eta_U}P(\eta_A,\eta_B,\eta_U,t)-\frac{\Phi_A\Phi_U\mu}{2\sqrt{\overline{V}}}\frac{\partial^3}{\partial\eta_A\partial\eta_U^2}P(\eta_A,\eta_B,\eta_U,t)+$$
$$\frac{\Phi_A\sqrt{\overline{V}}}{\frac{\Delta}{2}+\mu}\frac{\partial}{\partial\eta_A}P(\eta_A,\eta_B,\eta_U,t)-\frac{\Phi_A\sqrt{\overline{V}}}{\frac{\Delta}{2}+\mu}\frac{\partial}{\partial\eta_U}P(\eta_A,\eta_B,\eta_U,t)+$$
$$\Phi_A\eta_Bs\frac{\partial}{\partial\eta_A}P(\eta_A,\eta_B,\eta_U,t)+\Phi_A\eta_Bs\frac{\partial}{\partial\eta_B}P(\eta_A,\eta_B,\eta_U,t)-$$
$$2\Phi_A\eta_Bs\frac{\partial}{\partial\eta_U}P(\eta_A,\eta_B,\eta_U,t)-\Phi_A\eta_U\mu\frac{\partial}{\partial\eta_A}P(\eta_A,\eta_B,\eta_U,t)+$$
$$\Phi_A\eta_U\mu\frac{\partial}{\partial\eta_U}P(\eta_A,\eta_B,\eta_U,t)+\Phi_A\mu P(\eta_A,\eta_B,\eta_U,t)+\Phi_AsP(\eta_A,\eta_B,\eta_U,t)+$$
$$\frac{\Phi_A\frac{\partial^4}{\partial\eta_A^2\partial\eta_U^2}P(\eta_A,\eta_B,\eta_U,t)}{2\Delta\overline{V}+4\overline{V}\mu}-\frac{\Phi_A\frac{\partial^3}{\partial\eta_A^2\partial\eta_U}P(\eta_A,\eta_B,\eta_U,t)}{\Delta\sqrt{\overline{V}}+2\sqrt{\overline{V}}\mu}+\frac{\Phi_A\frac{\partial^3}{\partial\eta_A\partial\eta_U^2}P(\eta_A,\eta_B,\eta_U,t)}{\Delta\sqrt{\overline{V}}+2\sqrt{\overline{V}}\mu}+$$
$$\frac{\Phi_A\frac{\partial^2}{\partial\eta_A^2}P(\eta_A,\eta_B,\eta_U,t)}{2\left(\frac{\Delta}{2}+\mu\right)}-\frac{\Phi_A}{\frac{\Delta}{2}+\mu}\frac{\partial^2}{\partial\eta_A\partial\eta_U}P(\eta_A,\eta_B,\eta_U,t)+\frac{\Phi_A\frac{\partial^2}{\partial\eta_U^2}P(\eta_A,\eta_B,\eta_U,t)}{2\left(\frac{\Delta}{2}+\mu\right)}-$$
$$\frac{\Phi_A\eta_Bs}{2\overline{V}}\frac{\partial^3}{\partial\eta_A^2\partial\eta_U}P(\eta_A,\eta_B,\eta_U,t)+\frac{\Phi_A\eta_Bs}{2\overline{V}}\frac{\partial^3}{\partial\eta_A\partial\eta_U^2}P(\eta_A,\eta_B,\eta_U,t)-$$
$$\frac{\Phi_A\eta_Bs}{2\overline{V}}\frac{\partial^3}{\partial\eta_B^2\partial\eta_U}P(\eta_A,\eta_B,\eta_U,t)+\frac{\Phi_A\eta_Bs}{2\overline{V}}\frac{\partial^3}{\partial\eta_B\partial\eta_U^2}P(\eta_A,\eta_B,\eta_U,t)+$$

$$\frac{\Phi_A \eta_U \mu}{2V} \frac{\partial^3}{\partial \eta_A^2 \partial \eta_U} P(\eta_A, \eta_B, \eta_U, t) - \frac{\Phi_A \eta_U \mu}{2V} \frac{\partial^3}{\partial \eta_A \partial \eta_U^2} P(\eta_A, \eta_B, \eta_U, t) +$$
$$\frac{\Phi_A \mu}{2V} \frac{\partial^2}{\partial \eta_A^2} P(\eta_A, \eta_B, \eta_U, t) - \frac{\Phi_A \mu}{V} \frac{\partial^2}{\partial \eta_A \partial \eta_U} P(\eta_A, \eta_B, \eta_U, t) -$$
$$\frac{\Phi_A s}{V} \frac{\partial^2}{\partial \eta_B \partial \eta_U} P(\eta_A, \eta_B, \eta_U, t) + \frac{\Phi_A s}{2V} \frac{\partial^2}{\partial \eta_U^2} P(\eta_A, \eta_B, \eta_U, t) +$$
$$\frac{\Phi_A \eta_B s}{2\sqrt{V}} \frac{\partial^2}{\partial \eta_A^2} P(\eta_A, \eta_B, \eta_U, t) - \frac{\Phi_A \eta_B}{\sqrt{V}} s \frac{\partial^2}{\partial \eta_A \partial \eta_U} P(\eta_A, \eta_B, \eta_U, t) +$$
$$\frac{\Phi_A \eta_B s}{2\sqrt{V}} \frac{\partial^2}{\partial \eta_B^2} P(\eta_A, \eta_B, \eta_U, t) - \frac{\Phi_A \eta_B}{\sqrt{V}} s \frac{\partial^2}{\partial \eta_B \partial \eta_U} P(\eta_A, \eta_B, \eta_U, t) +$$
$$\frac{\Phi_A \eta_B}{\sqrt{V}} s \frac{\partial^2}{\partial \eta_U^2} P(\eta_A, \eta_B, \eta_U, t) + \frac{\Phi_A \eta_U \mu}{2\sqrt{V}} \frac{\partial^2}{\partial \eta_A^2} P(\eta_A, \eta_B, \eta_U, t) -$$
$$\frac{\Phi_A \eta_U}{\sqrt{V}} \mu \frac{\partial^2}{\partial \eta_A \partial \eta_U} P(\eta_A, \eta_B, \eta_U, t) + \frac{\Phi_A \eta_U \mu}{2\sqrt{V}} \frac{\partial^2}{\partial \eta_U^2} P(\eta_A, \eta_B, \eta_U, t) -$$
$$\frac{\Phi_A \mu}{\sqrt{V}} \frac{\partial}{\partial \eta_A} P(\eta_A, \eta_B, \eta_U, t) + \frac{\Phi_A \mu}{\sqrt{V}} \frac{\partial}{\partial \eta_U} P(\eta_A, \eta_B, \eta_U, t) + \frac{\Phi_A s}{\sqrt{V}} \frac{\partial}{\partial \eta_B} P(\eta_A, \eta_B, \eta_U, t) -$$
$$\frac{\Phi_A s}{\sqrt{V}} \frac{\partial}{\partial \eta_U} P(\eta_A, \eta_B, \eta_U, t) + \frac{\Phi_A \eta_B s}{4V^{\frac{3}{2}}} \frac{\partial^4}{\partial \eta_A^2 \partial \eta_U^2} P(\eta_A, \eta_B, \eta_U, t) +$$
$$\frac{\Phi_A \eta_B s}{4V^{\frac{3}{2}}} \frac{\partial^4}{\partial \eta_B^2 \partial \eta_U^2} P(\eta_A, \eta_B, \eta_U, t) + \frac{\Phi_A \eta_U \mu}{4V^{\frac{3}{2}}} \frac{\partial^4}{\partial \eta_A^2 \partial \eta_U^2} P(\eta_A, \eta_B, \eta_U, t) +$$
$$\frac{\Phi_A \mu}{2V^{\frac{3}{2}}} \frac{\partial^3}{\partial \eta_A^2 \partial \eta_U} P(\eta_A, \eta_B, \eta_U, t) + \frac{\Phi_A s}{2V^{\frac{3}{2}}} \frac{\partial^3}{\partial \eta_B \partial \eta_U^2} P(\eta_A, \eta_B, \eta_U, t) -$$
$$\Phi_B \Phi_U \sqrt{V} \mu \frac{\partial}{\partial \eta_B} P(\eta_A, \eta_B, \eta_U, t) + \Phi_B \Phi_U \sqrt{V} \mu \frac{\partial}{\partial \eta_U} P(\eta_A, \eta_B, \eta_U, t) +$$
$$\frac{\Phi_B \Phi_U}{2} \mu \frac{\partial^2}{\partial \eta_B^2} P(\eta_A, \eta_B, \eta_U, t) - \Phi_B \Phi_U \mu \frac{\partial^2}{\partial \eta_B \partial \eta_U} P(\eta_A, \eta_B, \eta_U, t) +$$
$$\frac{\Phi_B \Phi_U}{2} \mu \frac{\partial^2}{\partial \eta_U^2} P(\eta_A, \eta_B, \eta_U, t) + \frac{\Phi_B \Phi_U \mu}{4V} \frac{\partial^4}{\partial \eta_B^2 \partial \eta_U^2} P(\eta_A, \eta_B, \eta_U, t) +$$
$$\frac{\Phi_B \Phi_U \mu}{2\sqrt{V}} \frac{\partial^3}{\partial \eta_B^2 \partial \eta_U} P(\eta_A, \eta_B, \eta_U, t) - \frac{\Phi_B \Phi_U \mu}{2\sqrt{V}} \frac{\partial^3}{\partial \eta_B \partial \eta_U^2} P(\eta_A, \eta_B, \eta_U, t) +$$
$$\frac{\Phi_B \sqrt{V} \frac{\partial}{\partial \eta_B} P(\eta_A, \eta_B, \eta_U, t)}{-\frac{\Delta}{2} + \mu} - \frac{\Phi_B \sqrt{V} \frac{\partial}{\partial \eta_U} P(\eta_A, \eta_B, \eta_U, t)}{-\frac{\Delta}{2} + \mu} + \Phi_B \eta_A s \frac{\partial}{\partial \eta_A} P(\eta_A, \eta_B, \eta_U, t) +$$
$$\Phi_B \eta_A s \frac{\partial}{\partial \eta_B} P(\eta_A, \eta_B, \eta_U, t) - 2\Phi_B \eta_A s \frac{\partial}{\partial \eta_U} P(\eta_A, \eta_B, \eta_U, t) -$$
$$\Phi_B \eta_U \mu \frac{\partial}{\partial \eta_B} P(\eta_A, \eta_B, \eta_U, t) + \Phi_B \eta_U \mu \frac{\partial}{\partial \eta_U} P(\eta_A, \eta_B, \eta_U, t) +$$
$$\Phi_B \mu P(\eta_A, \eta_B, \eta_U, t) + \Phi_B s P(\eta_A, \eta_B, \eta_U, t) + \frac{\Phi_B \frac{\partial^4}{\partial \eta_B^2 \partial \eta_U^2} P(\eta_A, \eta_B, \eta_U, t)}{-2\Delta V + 4V\mu} -$$
$$\frac{\Phi_B \frac{\partial^3}{\partial \eta_B^2 \partial \eta_U} P(\eta_A, \eta_B, \eta_U, t)}{-\Delta \sqrt{V} + 2\sqrt{V}\mu} + \frac{\Phi_B \frac{\partial^3}{\partial \eta_B \partial \eta_U^2} P(\eta_A, \eta_B, \eta_U, t)}{-\Delta \sqrt{V} + 2\sqrt{V}\mu} + \frac{\Phi_B \frac{\partial^2}{\partial \eta_B^2} P(\eta_A, \eta_B, \eta_U, t)}{2\left(-\frac{\Delta}{2} + \mu\right)} -$$
$$\frac{\Phi_B \frac{\partial^2}{\partial \eta_B \partial \eta_U} P(\eta_A, \eta_B, \eta_U, t)}{-\frac{\Delta}{2} + \mu} + \frac{\Phi_B \frac{\partial^2}{\partial \eta_U^2} P(\eta_A, \eta_B, \eta_U, t)}{2\left(-\frac{\Delta}{2} + \mu\right)} - \frac{\Phi_B \eta_A s}{2V} \frac{\partial^3}{\partial \eta_A^2 \partial \eta_U} P(\eta_A, \eta_B, \eta_U, t) +$$
$$\frac{\Phi_B \eta_A s}{2V} \frac{\partial^3}{\partial \eta_A \partial \eta_U^2} P(\eta_A, \eta_B, \eta_U, t) - \frac{\Phi_B \eta_A s}{2V} \frac{\partial^3}{\partial \eta_B^2 \partial \eta_U} P(\eta_A, \eta_B, \eta_U, t) +$$
$$\frac{\Phi_B \eta_A s}{2V} \frac{\partial^3}{\partial \eta_B \partial \eta_U^2} P(\eta_A, \eta_B, \eta_U, t) + \frac{\Phi_B \eta_U \mu}{2V} \frac{\partial^3}{\partial \eta_B^2 \partial \eta_U} P(\eta_A, \eta_B, \eta_U, t) -$$
$$\frac{\Phi_B \eta_U \mu}{2V} \frac{\partial^3}{\partial \eta_B \partial \eta_U^2} P(\eta_A, \eta_B, \eta_U, t) + \frac{\Phi_B \mu}{2V} \frac{\partial^2}{\partial \eta_B^2} P(\eta_A, \eta_B, \eta_U, t) -$$
$$\frac{\Phi_B \mu}{V} \frac{\partial^2}{\partial \eta_B \partial \eta_U} P(\eta_A, \eta_B, \eta_U, t) - \frac{\Phi_B s}{V} \frac{\partial^2}{\partial \eta_A \partial \eta_U} P(\eta_A, \eta_B, \eta_U, t) +$$
$$\frac{\Phi_B s}{2V} \frac{\partial^2}{\partial \eta_U^2} P(\eta_A, \eta_B, \eta_U, t) + \frac{\Phi_B \eta_A s}{2\sqrt{V}} \frac{\partial^2}{\partial \eta_A^2} P(\eta_A, \eta_B, \eta_U, t) -$$

$$\frac{\Phi_B\eta_A}{\sqrt{V}}s\frac{\partial^2}{\partial\eta_A\partial\eta_U}P(\eta_A,\eta_B,\eta_U,t)+\frac{\Phi_B\eta_As}{2\sqrt{V}}\frac{\partial^2}{\partial\eta_B^2}P(\eta_A,\eta_B,\eta_U,t)-$$
$$\frac{\Phi_B\eta_A}{\sqrt{V}}s\frac{\partial^2}{\partial\eta_B\partial\eta_U}P(\eta_A,\eta_B,\eta_U,t)+\frac{\Phi_B\eta_A}{\sqrt{V}}s\frac{\partial^2}{\partial\eta_U^2}P(\eta_A,\eta_B,\eta_U,t)+$$
$$\frac{\Phi_B\eta_U\mu}{2\sqrt{V}}\frac{\partial^2}{\partial\eta_B^2}P(\eta_A,\eta_B,\eta_U,t)-\frac{\Phi_B\eta_U}{\sqrt{V}}\mu\frac{\partial^2}{\partial\eta_B\partial\eta_U}P(\eta_A,\eta_B,\eta_U,t)+$$
$$\frac{\Phi_B\eta_U\mu}{2\sqrt{V}}\frac{\partial^2}{\partial\eta_U^2}P(\eta_A,\eta_B,\eta_U,t)-\frac{\Phi_B\mu}{\sqrt{V}}\frac{\partial}{\partial\eta_B}P(\eta_A,\eta_B,\eta_U,t)+$$
$$\frac{\Phi_B\mu}{\sqrt{V}}\frac{\partial}{\partial\eta_U}P(\eta_A,\eta_B,\eta_U,t)+\frac{\Phi_Bs}{\sqrt{V}}\frac{\partial}{\partial\eta_A}P(\eta_A,\eta_B,\eta_U,t)-\frac{\Phi_Bs}{\sqrt{V}}\frac{\partial}{\partial\eta_U}P(\eta_A,\eta_B,\eta_U,t)+$$
$$\frac{\Phi_B\eta_As}{4V^{\frac{3}{2}}}\frac{\partial^4}{\partial\eta_A^2\partial\eta_U^2}P(\eta_A,\eta_B,\eta_U,t)+\frac{\Phi_B\eta_As}{4V^{\frac{3}{2}}}\frac{\partial^4}{\partial\eta_B^2\partial\eta_U^2}P(\eta_A,\eta_B,\eta_U,t)+$$
$$\frac{\Phi_B\eta_U\mu}{4V^{\frac{3}{2}}}\frac{\partial^4}{\partial\eta_B^2\partial\eta_U^2}P(\eta_A,\eta_B,\eta_U,t)+\frac{\Phi_B\mu}{2V^{\frac{3}{2}}}\frac{\partial^3}{\partial\eta_B^2\partial\eta_U}P(\eta_A,\eta_B,\eta_U,t)+$$
$$\frac{\Phi_Bs}{2V^{\frac{3}{2}}}\frac{\partial^3}{\partial\eta_A\partial\eta_U^2}P(\eta_A,\eta_B,\eta_U,t)-\Phi_U\sqrt{V}\mu\frac{\partial}{\partial\eta_A}P(\eta_A,\eta_B,\eta_U,t)-$$
$$\Phi_U\sqrt{V}\mu\frac{\partial}{\partial\eta_B}P(\eta_A,\eta_B,\eta_U,t)+2\Phi_U\sqrt{V}\mu\frac{\partial}{\partial\eta_U}P(\eta_A,\eta_B,\eta_U,t)-$$
$$\Phi_U\eta_A\mu\frac{\partial}{\partial\eta_A}P(\eta_A,\eta_B,\eta_U,t)+\Phi_U\eta_A\mu\frac{\partial}{\partial\eta_U}P(\eta_A,\eta_B,\eta_U,t)-$$
$$\Phi_U\eta_B\mu\frac{\partial}{\partial\eta_B}P(\eta_A,\eta_B,\eta_U,t)+\Phi_U\eta_B\mu\frac{\partial}{\partial\eta_U}P(\eta_A,\eta_B,\eta_U,t)-$$
$$2\Phi_U\mu P(\eta_A,\eta_B,\eta_U,t)+\frac{\Phi_U\mu}{2}\frac{\partial^2}{\partial\eta_A^2}P(\eta_A,\eta_B,\eta_U,t)-$$
$$\Phi_U\mu\frac{\partial^2}{\partial\eta_A\partial\eta_U}P(\eta_A,\eta_B,\eta_U,t)+\frac{\Phi_U\mu}{2}\frac{\partial^2}{\partial\eta_B^2}P(\eta_A,\eta_B,\eta_U,t)-$$
$$\Phi_U\mu\frac{\partial^2}{\partial\eta_B\partial\eta_U}P(\eta_A,\eta_B,\eta_U,t)+\Phi_U\mu\frac{\partial^2}{\partial\eta_U^2}P(\eta_A,\eta_B,\eta_U,t)+$$
$$\frac{\Phi_U\eta_A\mu}{2V}\frac{\partial^3}{\partial\eta_A^2\partial\eta_U}P(\eta_A,\eta_B,\eta_U,t)-\frac{\Phi_U\eta_A\mu}{2V}\frac{\partial^3}{\partial\eta_A\partial\eta_U^2}P(\eta_A,\eta_B,\eta_U,t)+$$
$$\frac{\Phi_U\eta_B\mu}{2V}\frac{\partial^3}{\partial\eta_B^2\partial\eta_U}P(\eta_A,\eta_B,\eta_U,t)-\frac{\Phi_U\eta_B\mu}{2V}\frac{\partial^3}{\partial\eta_B\partial\eta_U^2}P(\eta_A,\eta_B,\eta_U,t)+$$
$$\frac{\Phi_U\mu}{V}\frac{\partial^2}{\partial\eta_A\partial\eta_U}P(\eta_A,\eta_B,\eta_U,t)+\frac{\Phi_U\mu}{V}\frac{\partial^2}{\partial\eta_B\partial\eta_U}P(\eta_A,\eta_B,\eta_U,t)-$$
$$\frac{\Phi_U\mu}{V}\frac{\partial^2}{\partial\eta_U^2}P(\eta_A,\eta_B,\eta_U,t)+\frac{\Phi_U\mu}{4V}\frac{\partial^4}{\partial\eta_A^2\partial\eta_U^2}P(\eta_A,\eta_B,\eta_U,t)+$$
$$\frac{\Phi_U\mu}{4V}\frac{\partial^4}{\partial\eta_B^2\partial\eta_U^2}P(\eta_A,\eta_B,\eta_U,t)+\frac{\Phi_U\eta_A\mu}{2\sqrt{V}}\frac{\partial^2}{\partial\eta_A^2}P(\eta_A,\eta_B,\eta_U,t)-$$
$$\frac{\Phi_U\eta_A}{\sqrt{V}}\mu\frac{\partial^2}{\partial\eta_A\partial\eta_U}P(\eta_A,\eta_B,\eta_U,t)+\frac{\Phi_U\eta_A\mu}{2\sqrt{V}}\frac{\partial^2}{\partial\eta_U^2}P(\eta_A,\eta_B,\eta_U,t)+$$
$$\frac{\Phi_U\eta_B\mu}{2\sqrt{V}}\frac{\partial^2}{\partial\eta_B^2}P(\eta_A,\eta_B,\eta_U,t)-\frac{\Phi_U\eta_B}{\sqrt{V}}\mu\frac{\partial^2}{\partial\eta_B\partial\eta_U}P(\eta_A,\eta_B,\eta_U,t)+$$
$$\frac{\Phi_U\eta_B\mu}{2\sqrt{V}}\frac{\partial^2}{\partial\eta_U^2}P(\eta_A,\eta_B,\eta_U,t)+\frac{\Phi_U\mu}{\sqrt{V}}\frac{\partial}{\partial\eta_A}P(\eta_A,\eta_B,\eta_U,t)+$$
$$\frac{\Phi_U\mu}{\sqrt{V}}\frac{\partial}{\partial\eta_B}P(\eta_A,\eta_B,\eta_U,t)-\frac{2\Phi_U}{\sqrt{V}}\mu\frac{\partial}{\partial\eta_U}P(\eta_A,\eta_B,\eta_U,t)+$$
$$\frac{\Phi_U\mu}{2\sqrt{V}}\frac{\partial^3}{\partial\eta_A^2\partial\eta_U}P(\eta_A,\eta_B,\eta_U,t)-\frac{\Phi_U\mu}{2\sqrt{V}}\frac{\partial^3}{\partial\eta_A\partial\eta_U^2}P(\eta_A,\eta_B,\eta_U,t)+$$
$$\frac{\Phi_U\mu}{2\sqrt{V}}\frac{\partial^3}{\partial\eta_B^2\partial\eta_U}P(\eta_A,\eta_B,\eta_U,t)-\frac{\Phi_U\mu}{2\sqrt{V}}\frac{\partial^3}{\partial\eta_B\partial\eta_U^2}P(\eta_A,\eta_B,\eta_U,t)+$$
$$\frac{\Phi_U\eta_A\mu}{4V^{\frac{3}{2}}}\frac{\partial^4}{\partial\eta_A^2\partial\eta_U^2}P(\eta_A,\eta_B,\eta_U,t)+\frac{\Phi_U\eta_B\mu}{4V^{\frac{3}{2}}}\frac{\partial^4}{\partial\eta_B^2\partial\eta_U^2}P(\eta_A,\eta_B,\eta_U,t)+$$
$$\frac{\Phi_U\mu}{2V^{\frac{3}{2}}}\frac{\partial^3}{\partial\eta_A\partial\eta_U^2}P(\eta_A,\eta_B,\eta_U,t)+\frac{\Phi_U\mu}{2V^{\frac{3}{2}}}\frac{\partial^3}{\partial\eta_B\partial\eta_U^2}P(\eta_A,\eta_B,\eta_U,t)+$$

$$\frac{\eta_A \frac{\partial^4}{\partial\eta_A^2\partial\eta_U^2} P(\eta_A,\eta_B,\eta_U,t)}{2\Delta\overline{V}^{\frac{3}{2}}+4\overline{V}^{\frac{3}{2}}\mu} - \frac{\eta_A \frac{\partial^3}{\partial\eta_A^2\partial\eta_U} P(\eta_A,\eta_B,\eta_U,t)}{\Delta\overline{V}+2\overline{V}\mu} + \frac{\eta_A \frac{\partial^3}{\partial\eta_A\partial\eta_U^2} P(\eta_A,\eta_B,\eta_U,t)}{\Delta\overline{V}+2\overline{V}\mu} +$$

$$\frac{\eta_A \frac{\partial^2}{\partial\eta_A^2} P(\eta_A,\eta_B,\eta_U,t)}{\Delta\sqrt{\overline{V}}+2\sqrt{\overline{V}}\mu} + \frac{\eta_A \frac{\partial^2}{\partial\eta_U^2} P(\eta_A,\eta_B,\eta_U,t)}{\Delta\sqrt{\overline{V}}+2\sqrt{\overline{V}}\mu} - \frac{\eta_A \frac{\partial^2}{\partial\eta_A\partial\eta_U} P(\eta_A,\eta_B,\eta_U,t)}{\frac{\Delta\sqrt{\overline{V}}}{2}+\sqrt{\overline{V}}\mu} +$$

$$\frac{\eta_A}{\frac{\Delta}{2}+\mu}\frac{\partial}{\partial\eta_A}P(\eta_A,\eta_B,\eta_U,t) - \frac{\eta_A}{\frac{\Delta}{2}+\mu}\frac{\partial}{\partial\eta_U}P(\eta_A,\eta_B,\eta_U,t) + \frac{\eta_B \frac{\partial^4}{\partial\eta_B^2\partial\eta_U^2} P(\eta_A,\eta_B,\eta_U,t)}{-2\Delta\overline{V}^{\frac{3}{2}}+4\overline{V}^{\frac{3}{2}}\mu} -$$

$$\frac{\eta_B \frac{\partial^3}{\partial\eta_B^2\partial\eta_U} P(\eta_A,\eta_B,\eta_U,t)}{-\Delta\overline{V}+2\overline{V}\mu} + \frac{\eta_B \frac{\partial^3}{\partial\eta_B\partial\eta_U^2} P(\eta_A,\eta_B,\eta_U,t)}{-\Delta\overline{V}+2\overline{V}\mu} - \frac{\eta_B \frac{\partial^2}{\partial\eta_B\partial\eta_U} P(\eta_A,\eta_B,\eta_U,t)}{-\frac{\Delta\sqrt{\overline{V}}}{2}+\sqrt{\overline{V}}\mu} +$$

$$\frac{\eta_B \frac{\partial^2}{\partial\eta_B^2} P(\eta_A,\eta_B,\eta_U,t)}{-\Delta\sqrt{\overline{V}}+2\sqrt{\overline{V}}\mu} + \frac{\eta_B \frac{\partial^2}{\partial\eta_U^2} P(\eta_A,\eta_B,\eta_U,t)}{-\Delta\sqrt{\overline{V}}+2\sqrt{\overline{V}}\mu} + \frac{\eta_B \frac{\partial}{\partial\eta_B} P(\eta_A,\eta_B,\eta_U,t)}{-\frac{\Delta}{2}+\mu} -$$

$$\frac{\eta_B \frac{\partial}{\partial\eta_U} P(\eta_A,\eta_B,\eta_U,t)}{-\frac{\Delta}{2}+\mu} - \eta_U\mu\frac{\partial}{\partial\eta_A}P(\eta_A,\eta_B,\eta_U,t) - \eta_U\mu\frac{\partial}{\partial\eta_B}P(\eta_A,\eta_B,\eta_U,t) +$$

$$2\eta_U\mu\frac{\partial}{\partial\eta_U}P(\eta_A,\eta_B,\eta_U,t) + 2\mu P(\eta_A,\eta_B,\eta_U,t) + \frac{\frac{\partial^3}{\partial\eta_A\partial\eta_U^2} P(\eta_A,\eta_B,\eta_U,t)}{\Delta\overline{V}^{\frac{3}{2}}+2\overline{V}^{\frac{3}{2}}\mu} +$$

$$\frac{\frac{\partial^3}{\partial\eta_B\partial\eta_U^2} P(\eta_A,\eta_B,\eta_U,t)}{-\Delta\overline{V}^{\frac{3}{2}}+2\overline{V}^{\frac{3}{2}}\mu} + \frac{\frac{\partial^2}{\partial\eta_U^2} P(\eta_A,\eta_B,\eta_U,t)}{\Delta\overline{V}+2\overline{V}\mu} - \frac{\frac{\partial^2}{\partial\eta_A\partial\eta_U} P(\eta_A,\eta_B,\eta_U,t)}{\frac{\Delta\overline{V}}{2}+\overline{V}\mu} -$$

$$\frac{\frac{\partial^2}{\partial\eta_B\partial\eta_U} P(\eta_A,\eta_B,\eta_U,t)}{-\frac{\Delta\overline{V}}{2}+\overline{V}\mu} + \frac{\frac{\partial^2}{\partial\eta_U^2} P(\eta_A,\eta_B,\eta_U,t)}{-\Delta\overline{V}+2\overline{V}\mu} + \frac{\frac{\partial}{\partial\eta_A} P(\eta_A,\eta_B,\eta_U,t)}{\frac{\Delta\sqrt{\overline{V}}}{2}+\sqrt{\overline{V}}\mu} -$$

$$\frac{\frac{\partial}{\partial\eta_U} P(\eta_A,\eta_B,\eta_U,t)}{\frac{\Delta\sqrt{\overline{V}}}{2}+\sqrt{\overline{V}}\mu} + \frac{\frac{\partial}{\partial\eta_B} P(\eta_A,\eta_B,\eta_U,t)}{-\frac{\Delta\sqrt{\overline{V}}}{2}+\sqrt{\overline{V}}\mu} - \frac{\frac{\partial}{\partial\eta_U} P(\eta_A,\eta_B,\eta_U,t)}{-\frac{\Delta\sqrt{\overline{V}}}{2}+\sqrt{\overline{V}}\mu} + \frac{P(\eta_A,\eta_B,\eta_U,t)}{\frac{\Delta}{2}+\mu} +$$

$$\frac{P(\eta_A,\eta_B,\eta_U,t)}{-\frac{\Delta}{2}+\mu} + \frac{\eta_A\eta_B s}{2\overline{V}}\frac{\partial^2}{\partial\eta_A^2}P(\eta_A,\eta_B,\eta_U,t) - \frac{\eta_A\eta_B}{\overline{V}}s\frac{\partial^2}{\partial\eta_A\partial\eta_U}P(\eta_A,\eta_B,\eta_U,t) +$$

$$\frac{\eta_A\eta_B s}{2\overline{V}}\frac{\partial^2}{\partial\eta_B^2}P(\eta_A,\eta_B,\eta_U,t) - \frac{\eta_A\eta_B}{\overline{V}}s\frac{\partial^2}{\partial\eta_B\partial\eta_U}P(\eta_A,\eta_B,\eta_U,t) +$$

$$\frac{\eta_A\eta_B}{\overline{V}}s\frac{\partial^2}{\partial\eta_U^2}P(\eta_A,\eta_B,\eta_U,t) + \frac{\eta_A\eta_U\mu}{2\overline{V}}\frac{\partial^2}{\partial\eta_A^2}P(\eta_A,\eta_B,\eta_U,t) -$$

$$\frac{\eta_A\eta_U}{\overline{V}}\mu\frac{\partial^2}{\partial\eta_A\partial\eta_U}P(\eta_A,\eta_B,\eta_U,t) + \frac{\eta_A\eta_U\mu}{2\overline{V}}\frac{\partial^2}{\partial\eta_U^2}P(\eta_A,\eta_B,\eta_U,t) -$$

$$\frac{\eta_A\mu}{\overline{V}}\frac{\partial}{\partial\eta_A}P(\eta_A,\eta_B,\eta_U,t) + \frac{\eta_A\mu}{\overline{V}}\frac{\partial}{\partial\eta_U}P(\eta_A,\eta_B,\eta_U,t) + \frac{\eta_A s}{\overline{V}}\frac{\partial}{\partial\eta_B}P(\eta_A,\eta_B,\eta_U,t) -$$

$$\frac{\eta_A s}{\overline{V}}\frac{\partial}{\partial\eta_U}P(\eta_A,\eta_B,\eta_U,t) + \frac{\eta_B\eta_U\mu}{2\overline{V}}\frac{\partial^2}{\partial\eta_B^2}P(\eta_A,\eta_B,\eta_U,t) -$$

$$\frac{\eta_B\eta_U}{\overline{V}}\mu\frac{\partial^2}{\partial\eta_B\partial\eta_U}P(\eta_A,\eta_B,\eta_U,t) + \frac{\eta_B\eta_U\mu}{2\overline{V}}\frac{\partial^2}{\partial\eta_U^2}P(\eta_A,\eta_B,\eta_U,t) -$$

$$\frac{\eta_B\mu}{\overline{V}}\frac{\partial}{\partial\eta_B}P(\eta_A,\eta_B,\eta_U,t) + \frac{\eta_B\mu}{\overline{V}}\frac{\partial}{\partial\eta_U}P(\eta_A,\eta_B,\eta_U,t) + \frac{\eta_B s}{\overline{V}}\frac{\partial}{\partial\eta_A}P(\eta_A,\eta_B,\eta_U,t) -$$

$$\frac{\eta_B s}{\overline{V}}\frac{\partial}{\partial\eta_U}P(\eta_A,\eta_B,\eta_U,t) + \frac{\eta_U\mu}{\overline{V}}\frac{\partial}{\partial\eta_A}P(\eta_A,\eta_B,\eta_U,t) + \frac{\eta_U\mu}{\overline{V}}\frac{\partial}{\partial\eta_B}P(\eta_A,\eta_B,\eta_U,t) -$$

$$\frac{2\eta_U}{\overline{V}}\mu\frac{\partial}{\partial\eta_U}P(\eta_A,\eta_B,\eta_U,t) + \frac{\eta_U\mu}{2\overline{V}}\frac{\partial^3}{\partial\eta_A^2\partial\eta_U}P(\eta_A,\eta_B,\eta_U,t) -$$

$$\frac{\eta_U\mu}{2\overline{V}}\frac{\partial^3}{\partial\eta_A\partial\eta_U^2}P(\eta_A,\eta_B,\eta_U,t) + \frac{\eta_U\mu}{2\overline{V}}\frac{\partial^3}{\partial\eta_B^2\partial\eta_U}P(\eta_A,\eta_B,\eta_U,t) -$$

$$\frac{\eta_U\mu}{2\overline{V}}\frac{\partial^3}{\partial\eta_B\partial\eta_U^2}P(\eta_A,\eta_B,\eta_U,t) - \frac{2\mu}{\overline{V}}P(\eta_A,\eta_B,\eta_U,t) + \frac{\mu}{2\overline{V}}\frac{\partial^2}{\partial\eta_A^2}P(\eta_A,\eta_B,\eta_U,t) -$$

$$\frac{\mu}{\overline{V}}\frac{\partial^2}{\partial\eta_A\partial\eta_U}P(\eta_A,\eta_B,\eta_U,t) + \frac{\mu}{2\overline{V}}\frac{\partial^2}{\partial\eta_B^2}P(\eta_A,\eta_B,\eta_U,t) -$$

$$\frac{\mu}{\overline{V}}\frac{\partial^2}{\partial\eta_B\partial\eta_U}P(\eta_A,\eta_B,\eta_U,t)+\frac{\eta_A\eta_B s}{4\overline{V}^2}\frac{\partial^4}{\partial\eta_A^2\partial\eta_U^2}P(\eta_A,\eta_B,\eta_U,t)+$$
$$\frac{\eta_A\eta_B s}{4\overline{V}^2}\frac{\partial^4}{\partial\eta_B^2\partial\eta_U^2}P(\eta_A,\eta_B,\eta_U,t)+\frac{\eta_A\eta_U\mu}{4\overline{V}^2}\frac{\partial^4}{\partial\eta_A^2\partial\eta_U^2}P(\eta_A,\eta_B,\eta_U,t)+$$
$$\frac{\eta_A\mu}{2\overline{V}^2}\frac{\partial^3}{\partial\eta_A^2\partial\eta_U}P(\eta_A,\eta_B,\eta_U,t)+\frac{\eta_A s}{2\overline{V}^2}\frac{\partial^3}{\partial\eta_B\partial\eta_U^2}P(\eta_A,\eta_B,\eta_U,t)+$$
$$\frac{\eta_B\eta_U\mu}{4\overline{V}^2}\frac{\partial^4}{\partial\eta_B^2\partial\eta_U^2}P(\eta_A,\eta_B,\eta_U,t)+\frac{\eta_B\mu}{2\overline{V}^2}\frac{\partial^3}{\partial\eta_B^2\partial\eta_U}P(\eta_A,\eta_B,\eta_U,t)+$$
$$\frac{\eta_B s}{2\overline{V}^2}\frac{\partial^3}{\partial\eta_A\partial\eta_U^2}P(\eta_A,\eta_B,\eta_U,t)+\frac{\eta_U\mu}{2\overline{V}^2}\frac{\partial^3}{\partial\eta_A\partial\eta_U^2}P(\eta_A,\eta_B,\eta_U,t)+$$
$$\frac{\eta_U\mu}{2\overline{V}^2}\frac{\partial^3}{\partial\eta_B\partial\eta_U^2}P(\eta_A,\eta_B,\eta_U,t)+\frac{\mu}{\overline{V}^2}\frac{\partial^2}{\partial\eta_A\partial\eta_U}P(\eta_A,\eta_B,\eta_U,t)+$$
$$\frac{\mu}{\overline{V}^2}\frac{\partial^2}{\partial\eta_B\partial\eta_U}P(\eta_A,\eta_B,\eta_U,t)+\frac{\eta_A\eta_B}{\sqrt{\overline{V}}}s\frac{\partial}{\partial\eta_A}P(\eta_A,\eta_B,\eta_U,t)+$$
$$\frac{\eta_A\eta_B}{\sqrt{\overline{V}}}s\frac{\partial}{\partial\eta_B}P(\eta_A,\eta_B,\eta_U,t)-\frac{2\eta_A}{\sqrt{\overline{V}}}\eta_B s\frac{\partial}{\partial\eta_U}P(\eta_A,\eta_B,\eta_U,t)-$$
$$\frac{\eta_A\eta_U}{\sqrt{\overline{V}}}\mu\frac{\partial}{\partial\eta_A}P(\eta_A,\eta_B,\eta_U,t)+\frac{\eta_A\eta_U}{\sqrt{\overline{V}}}\mu\frac{\partial}{\partial\eta_U}P(\eta_A,\eta_B,\eta_U,t)+$$
$$\frac{\eta_A\mu}{\sqrt{\overline{V}}}P(\eta_A,\eta_B,\eta_U,t)+\frac{\eta_A s}{\sqrt{\overline{V}}}P(\eta_A,\eta_B,\eta_U,t)-\frac{\eta_B\eta_U}{\sqrt{\overline{V}}}\mu\frac{\partial}{\partial\eta_B}P(\eta_A,\eta_B,\eta_U,t)+$$
$$\frac{\eta_B\eta_U}{\sqrt{\overline{V}}}\mu\frac{\partial}{\partial\eta_U}P(\eta_A,\eta_B,\eta_U,t)+\frac{\eta_B\mu}{\sqrt{\overline{V}}}P(\eta_A,\eta_B,\eta_U,t)+\frac{\eta_B s}{\sqrt{\overline{V}}}P(\eta_A,\eta_B,\eta_U,t)-$$
$$\frac{2\eta_U}{\sqrt{\overline{V}}}\mu P(\eta_A,\eta_B,\eta_U,t)+\frac{\eta_U\mu}{2\sqrt{\overline{V}}}\frac{\partial^2}{\partial\eta_A^2}P(\eta_A,\eta_B,\eta_U,t)-$$
$$\frac{\eta_U\mu}{\sqrt{\overline{V}}}\frac{\partial^2}{\partial\eta_A\partial\eta_U}P(\eta_A,\eta_B,\eta_U,t)+\frac{\eta_U\mu}{2\sqrt{\overline{V}}}\frac{\partial^2}{\partial\eta_B^2}P(\eta_A,\eta_B,\eta_U,t)-$$
$$\frac{\eta_U\mu}{\sqrt{\overline{V}}}\frac{\partial^2}{\partial\eta_B\partial\eta_U}P(\eta_A,\eta_B,\eta_U,t)+\frac{\eta_U\mu}{\sqrt{\overline{V}}}\frac{\partial^2}{\partial\eta_U^2}P(\eta_A,\eta_B,\eta_U,t)-$$
$$\frac{\mu}{\sqrt{\overline{V}}}\frac{\partial}{\partial\eta_A}P(\eta_A,\eta_B,\eta_U,t)-\frac{\mu}{\sqrt{\overline{V}}}\frac{\partial}{\partial\eta_B}P(\eta_A,\eta_B,\eta_U,t)+$$
$$\frac{2\mu}{\sqrt{\overline{V}}}\frac{\partial}{\partial\eta_U}P(\eta_A,\eta_B,\eta_U,t)-\frac{\eta_A\eta_B s}{2\overline{V}^{\frac{3}{2}}}\frac{\partial^3}{\partial\eta_A^2\partial\eta_U}P(\eta_A,\eta_B,\eta_U,t)+$$
$$\frac{\eta_A\eta_B s}{2\overline{V}^{\frac{3}{2}}}\frac{\partial^3}{\partial\eta_A\partial\eta_U^2}P(\eta_A,\eta_B,\eta_U,t)-\frac{\eta_A\eta_B s}{2\overline{V}^{\frac{3}{2}}}\frac{\partial^3}{\partial\eta_B^2\partial\eta_U}P(\eta_A,\eta_B,\eta_U,t)+$$
$$\frac{\eta_A\eta_B s}{2\overline{V}^{\frac{3}{2}}}\frac{\partial^3}{\partial\eta_B\partial\eta_U^2}P(\eta_A,\eta_B,\eta_U,t)+\frac{\eta_A\eta_U\mu}{2\overline{V}^{\frac{3}{2}}}\frac{\partial^3}{\partial\eta_A^2\partial\eta_U}P(\eta_A,\eta_B,\eta_U,t)-$$
$$\frac{\eta_A\eta_U\mu}{2\overline{V}^{\frac{3}{2}}}\frac{\partial^3}{\partial\eta_A\partial\eta_U^2}P(\eta_A,\eta_B,\eta_U,t)+\frac{\eta_A\mu}{2\overline{V}^{\frac{3}{2}}}\frac{\partial^2}{\partial\eta_A^2}P(\eta_A,\eta_B,\eta_U,t)-$$
$$\frac{\eta_A\mu}{\overline{V}^{\frac{3}{2}}}\frac{\partial^2}{\partial\eta_A\partial\eta_U}P(\eta_A,\eta_B,\eta_U,t)-\frac{\eta_A s}{\overline{V}^{\frac{3}{2}}}\frac{\partial^2}{\partial\eta_B\partial\eta_U}P(\eta_A,\eta_B,\eta_U,t)+$$
$$\frac{\eta_A s}{2\overline{V}^{\frac{3}{2}}}\frac{\partial^2}{\partial\eta_U^2}P(\eta_A,\eta_B,\eta_U,t)+\frac{\eta_B\eta_U\mu}{2\overline{V}^{\frac{3}{2}}}\frac{\partial^3}{\partial\eta_B^2\partial\eta_U}P(\eta_A,\eta_B,\eta_U,t)-$$
$$\frac{\eta_B\eta_U\mu}{2\overline{V}^{\frac{3}{2}}}\frac{\partial^3}{\partial\eta_B\partial\eta_U^2}P(\eta_A,\eta_B,\eta_U,t)+\frac{\eta_B\mu}{2\overline{V}^{\frac{3}{2}}}\frac{\partial^2}{\partial\eta_B^2}P(\eta_A,\eta_B,\eta_U,t)-$$
$$\frac{\eta_B\mu}{\overline{V}^{\frac{3}{2}}}\frac{\partial^2}{\partial\eta_B\partial\eta_U}P(\eta_A,\eta_B,\eta_U,t)-\frac{\eta_B s}{\overline{V}^{\frac{3}{2}}}\frac{\partial^2}{\partial\eta_A\partial\eta_U}P(\eta_A,\eta_B,\eta_U,t)+$$
$$\frac{\eta_B s}{2\overline{V}^{\frac{3}{2}}}\frac{\partial^2}{\partial\eta_U^2}P(\eta_A,\eta_B,\eta_U,t)+\frac{\eta_U\mu}{\overline{V}^{\frac{3}{2}}}\frac{\partial^2}{\partial\eta_A\partial\eta_U}P(\eta_A,\eta_B,\eta_U,t)+$$
$$\frac{\eta_U\mu}{\overline{V}^{\frac{3}{2}}}\frac{\partial^2}{\partial\eta_B\partial\eta_U}P(\eta_A,\eta_B,\eta_U,t)-\frac{\eta_U\mu}{\overline{V}^{\frac{3}{2}}}\frac{\partial^2}{\partial\eta_U^2}P(\eta_A,\eta_B,\eta_U,t)+$$
$$\frac{\eta_U\mu}{4\overline{V}^{\frac{3}{2}}}\frac{\partial^4}{\partial\eta_A^2\partial\eta_U^2}P(\eta_A,\eta_B,\eta_U,t)+\frac{\eta_U\mu}{4\overline{V}^{\frac{3}{2}}}\frac{\partial^4}{\partial\eta_B^2\partial\eta_U^2}P(\eta_A,\eta_B,\eta_U,t)+$$

$$\frac{\mu}{\overline{V}^{\frac{3}{2}}}\frac{\partial}{\partial \eta_A}P(\eta_A,\eta_B,\eta_U,t)+\frac{\mu}{\overline{V}^{\frac{3}{2}}}\frac{\partial}{\partial \eta_B}P(\eta_A,\eta_B,\eta_U,t)-\frac{2\mu}{\overline{V}^{\frac{3}{2}}}\frac{\partial}{\partial \eta_U}P(\eta_A,\eta_B,\eta_U,t)+\frac{\mu}{2\overline{V}^{\frac{3}{2}}}\frac{\partial^3}{\partial \eta_A^2\partial \eta_U}P(\eta_A,\eta_B,\eta_U,t)+\frac{\mu}{2\overline{V}^{\frac{3}{2}}}\frac{\partial^3}{\partial \eta_B^2\partial \eta_U}P(\eta_A,\eta_B,\eta_U,t)$$

using substitution $U := -A - B + N$.

3. Fokker-Planck equation for stop-signal model of Seeley *et al.*

$$\frac{\partial}{\partial t}P(\eta_A,\eta_B,\eta_U,t) := \frac{1}{\overline{V}^{11}(\Delta^2-4\mu^2)}\Big(\frac{\Phi_A\Phi_U}{4}\Delta^3\overline{V}^{11}\frac{\partial^2}{\partial\eta_A^2}P(\eta_A,\eta_B,\eta_U,t)-\frac{\Phi_A\Phi_U}{2}\Delta^3\overline{V}^{11}\frac{\partial^2}{\partial\eta_A\partial\eta_U}P(\eta_A,\eta_B,\eta_U,t)+\frac{\Phi_A\Phi_U}{4}\Delta^3\overline{V}^{11}\frac{\partial^2}{\partial\eta_U^2}P(\eta_A,\eta_B,\eta_U,t)-\frac{\Phi_A\eta_U}{2}\Delta^3\overline{V}^{11}\frac{\partial}{\partial\eta_A}P(\eta_A,\eta_B,\eta_U,t)+\frac{\Phi_A\eta_U}{2}\Delta^3\overline{V}^{11}\frac{\partial}{\partial\eta_U}P(\eta_A,\eta_B,\eta_U,t)+\frac{\Delta^3\Phi_A}{2}\overline{V}^{11}P(\eta_A,\eta_B,\eta_U,t)-\frac{\Phi_B\Phi_U}{4}\Delta^3\overline{V}^{11}\frac{\partial^2}{\partial\eta_B^2}P(\eta_A,\eta_B,\eta_U,t)+\frac{\Phi_B\Phi_U}{2}\Delta^3\overline{V}^{11}\frac{\partial^2}{\partial\eta_B\partial\eta_U}P(\eta_A,\eta_B,\eta_U,t)-\frac{\Phi_B\Phi_U}{4}\Delta^3\overline{V}^{11}\frac{\partial^2}{\partial\eta_U^2}P(\eta_A,\eta_B,\eta_U,t)+\frac{\Phi_B\eta_U}{2}\Delta^3\overline{V}^{11}\frac{\partial}{\partial\eta_B}P(\eta_A,\eta_B,\eta_U,t)-\frac{\Phi_B\eta_U}{2}\Delta^3\overline{V}^{11}\frac{\partial}{\partial\eta_U}P(\eta_A,\eta_B,\eta_U,t)-\frac{\Delta^3\Phi_B}{2}\overline{V}^{11}P(\eta_A,\eta_B,\eta_U,t)-\frac{\Phi_U\eta_A}{2}\Delta^3\overline{V}^{11}\frac{\partial}{\partial\eta_A}P(\eta_A,\eta_B,\eta_U,t)+\frac{\Phi_U\eta_A}{2}\Delta^3\overline{V}^{11}\frac{\partial}{\partial\eta_U}P(\eta_A,\eta_B,\eta_U,t)+\frac{\Phi_U\eta_B}{2}\Delta^3\overline{V}^{11}\frac{\partial}{\partial\eta_B}P(\eta_A,\eta_B,\eta_U,t)-\frac{\Phi_U\eta_B}{2}\Delta^3\overline{V}^{11}\frac{\partial}{\partial\eta_U}P(\eta_A,\eta_B,\eta_U,t)+\frac{\Delta^3\Phi_U}{4}\overline{V}^{11}\frac{\partial^2}{\partial\eta_A^2}P(\eta_A,\eta_B,\eta_U,t)-\frac{\Delta^3\Phi_U}{2}\overline{V}^{11}\frac{\partial^2}{\partial\eta_A\partial\eta_U}P(\eta_A,\eta_B,\eta_U,t)-\frac{\Delta^3\Phi_U}{4}\overline{V}^{11}\frac{\partial^2}{\partial\eta_B^2}P(\eta_A,\eta_B,\eta_U,t)+\frac{\Delta^3\Phi_U}{2}\overline{V}^{11}\frac{\partial^2}{\partial\eta_B\partial\eta_U}P(\eta_A,\eta_B,\eta_U,t)-\frac{\Delta^3\eta_U}{2}\overline{V}^{11}\frac{\partial}{\partial\eta_A}P(\eta_A,\eta_B,\eta_U,t)+\frac{\Delta^3\eta_U}{2}\overline{V}^{11}\frac{\partial}{\partial\eta_B}P(\eta_A,\eta_B,\eta_U,t)+\frac{\Phi_A\Phi_B}{2}\Delta^2\overline{V}^{11}s\frac{\partial^2}{\partial\eta_A^2}P(\eta_A,\eta_B,\eta_U,t)-\Delta^2\Phi_A\Phi_B\overline{V}^{11}s\frac{\partial^2}{\partial\eta_A\partial\eta_U}P(\eta_A,\eta_B,\eta_U,t)+\frac{\Phi_A\Phi_B}{2}\Delta^2\overline{V}^{11}s\frac{\partial^2}{\partial\eta_B^2}P(\eta_A,\eta_B,\eta_U,t)-\Delta^2\Phi_A\Phi_B\overline{V}^{11}s\frac{\partial^2}{\partial\eta_B\partial\eta_U}P(\eta_A,\eta_B,\eta_U,t)+\Delta^2\Phi_A\Phi_B\overline{V}^{11}s\frac{\partial^2}{\partial\eta_U^2}P(\eta_A,\eta_B,\eta_U,t)+\frac{\Phi_A\Phi_U}{2}\Delta^2\overline{V}^{11}\mu\frac{\partial^2}{\partial\eta_A^2}P(\eta_A,\eta_B,\eta_U,t)-\Delta^2\Phi_A\Phi_U\overline{V}^{11}\mu\frac{\partial^2}{\partial\eta_A\partial\eta_U}P(\eta_A,\eta_B,\eta_U,t)+\frac{\Phi_A\Phi_U}{2}\Delta^2\overline{V}^{11}\mu\frac{\partial^2}{\partial\eta_U^2}P(\eta_A,\eta_B,\eta_U,t)+\Delta^2\Phi_A\overline{V}^{11}\eta_Bs\frac{\partial}{\partial\eta_A}P(\eta_A,\eta_B,\eta_U,t)+\Delta^2\Phi_A\overline{V}^{11}\eta_Bs\frac{\partial}{\partial\eta_B}P(\eta_A,\eta_B,\eta_U,t)-2\Delta^2\Phi_A\overline{V}^{11}\eta_Bs\frac{\partial}{\partial\eta_U}P(\eta_A,\eta_B,\eta_U,t)-\Delta^2\Phi_A\overline{V}^{11}\eta_U\mu\frac{\partial}{\partial\eta_A}P(\eta_A,\eta_B,\eta_U,t)+\Delta^2\Phi_A\overline{V}^{11}\eta_U\mu\frac{\partial}{\partial\eta_U}P(\eta_A,\eta_B,\eta_U,t)+\Delta^2\Phi_A\overline{V}^{11}\mu P(\eta_A,\eta_B,\eta_U,t)+\Delta^2\Phi_A\overline{V}^{11}sP(\eta_A,\eta_B,\eta_U,t)+\frac{\Phi_B\Phi_U}{2}\Delta^2\overline{V}^{11}\mu\frac{\partial^2}{\partial\eta_B^2}P(\eta_A,\eta_B,\eta_U,t)-\Delta^2\Phi_B\Phi_U\overline{V}^{11}\mu\frac{\partial^2}{\partial\eta_B\partial\eta_U}P(\eta_A,\eta_B,\eta_U,t)+\frac{\Phi_B\Phi_U}{2}\Delta^2\overline{V}^{11}\mu\frac{\partial^2}{\partial\eta_U^2}P(\eta_A,\eta_B,\eta_U,t)+\Delta^2\Phi_B\overline{V}^{11}\eta_As\frac{\partial}{\partial\eta_A}P(\eta_A,\eta_B,\eta_U,t)+\Delta^2\Phi_B\overline{V}^{11}\eta_As\frac{\partial}{\partial\eta_B}P(\eta_A,\eta_B,\eta_U,t)-2\Delta^2\Phi_B\overline{V}^{11}\eta_As\frac{\partial}{\partial\eta_U}P(\eta_A,\eta_B,\eta_U,t)-\Delta^2\Phi_B\overline{V}^{11}\eta_U\mu\frac{\partial}{\partial\eta_B}P(\eta_A,\eta_B,\eta_U,t)+\Delta^2\Phi_B\overline{V}^{11}\eta_U\mu\frac{\partial}{\partial\eta_U}P(\eta_A,\eta_B,\eta_U,t)+\Delta^2\Phi_B\overline{V}^{11}\mu P(\eta_A,\eta_B,\eta_U,t)+\Delta^2\Phi_B\overline{V}^{11}sP(\eta_A,\eta_B,\eta_U,t)-\Delta^2\Phi_U\overline{V}^{11}\eta_A\mu\frac{\partial}{\partial\eta_A}P(\eta_A,\eta_B,\eta_U,t)+\Delta^2\Phi_U\overline{V}^{11}\eta_A\mu\frac{\partial}{\partial\eta_U}P(\eta_A,\eta_B,\eta_U,t)-$$

$\Delta^2\Phi_U\overline{V}^{11}\eta_B\mu\frac{\partial}{\partial\eta_B}P(\eta_A,\eta_B,\eta_U,t) + \Delta^2\Phi_U\overline{V}^{11}\eta_B\mu\frac{\partial}{\partial\eta_U}P(\eta_A,\eta_B,\eta_U,t) -$
$2\Delta^2\Phi_U\overline{V}^{11}\mu P(\eta_A,\eta_B,\eta_U,t) + \frac{\Phi_U\mu}{2}\Delta^2\overline{V}^{11}\frac{\partial^2}{\partial\eta_A^2}P(\eta_A,\eta_B,\eta_U,t) -$
$\Delta^2\Phi_U\overline{V}^{11}\mu\frac{\partial^2}{\partial\eta_A\partial\eta_U}P(\eta_A,\eta_B,\eta_U,t) + \frac{\Phi_U\mu}{2}\Delta^2\overline{V}^{11}\frac{\partial^2}{\partial\eta_B^2}P(\eta_A,\eta_B,\eta_U,t) -$
$\Delta^2\Phi_U\overline{V}^{11}\mu\frac{\partial^2}{\partial\eta_B\partial\eta_U}P(\eta_A,\eta_B,\eta_U,t) + \Delta^2\Phi_U\overline{V}^{11}\mu\frac{\partial^2}{\partial\eta_U^2}P(\eta_A,\eta_B,\eta_U,t) -$
$\Delta^2\overline{V}^{11}\eta_U\mu\frac{\partial}{\partial\eta_A}P(\eta_A,\eta_B,\eta_U,t) - \Delta^2\overline{V}^{11}\eta_U\mu\frac{\partial}{\partial\eta_B}P(\eta_A,\eta_B,\eta_U,t) +$
$2\Delta^2\overline{V}^{11}\eta_U\mu\frac{\partial}{\partial\eta_U}P(\eta_A,\eta_B,\eta_U,t) + 2\Delta^2\overline{V}^{11}\mu P(\eta_A,\eta_B,\eta_U,t) -$
$\Delta\Phi_A\Phi_U\overline{V}^{11}\mu^2\frac{\partial^2}{\partial\eta_A^2}P(\eta_A,\eta_B,\eta_U,t) + 2\Delta\Phi_A\Phi_U\overline{V}^{11}\mu^2\frac{\partial^2}{\partial\eta_A\partial\eta_U}P(\eta_A,\eta_B,\eta_U,t) -$
$\Delta\Phi_A\Phi_U\overline{V}^{11}\mu^2\frac{\partial^2}{\partial\eta_U^2}P(\eta_A,\eta_B,\eta_U,t) - \Delta\Phi_A\overline{V}^{\frac{21}{2}}\frac{\partial^3}{\partial\eta_A^2\partial\eta_U}P(\eta_A,\eta_B,\eta_U,t) +$
$\Delta\Phi_A\overline{V}^{\frac{21}{2}}\frac{\partial^3}{\partial\eta_A\partial\eta_U^2}P(\eta_A,\eta_B,\eta_U,t) + 2\Delta\Phi_A\overline{V}^{11}\eta_U\mu^2\frac{\partial}{\partial\eta_A}P(\eta_A,\eta_B,\eta_U,t) -$
$2\Delta\Phi_A\overline{V}^{11}\eta_U\mu^2\frac{\partial}{\partial\eta_U}P(\eta_A,\eta_B,\eta_U,t) - 2\Delta\Phi_A\overline{V}^{11}\mu^2 P(\eta_A,\eta_B,\eta_U,t) +$
$\Delta\Phi_A\overline{V}^{11}\frac{\partial^2}{\partial\eta_A^2}P(\eta_A,\eta_B,\eta_U,t) - 2\Delta\Phi_A\overline{V}^{11}\frac{\partial^2}{\partial\eta_A\partial\eta_U}P(\eta_A,\eta_B,\eta_U,t) +$
$\Delta\Phi_A\overline{V}^{11}\frac{\partial^2}{\partial\eta_U^2}P(\eta_A,\eta_B,\eta_U,t) + \frac{\Delta\Phi_A}{2}\overline{V}^{10}\frac{\partial^4}{\partial\eta_A^2\partial\eta_U^2}P(\eta_A,\eta_B,\eta_U,t) +$
$\Delta\Phi_B\Phi_U\overline{V}^{11}\mu^2\frac{\partial^2}{\partial\eta_B^2}P(\eta_A,\eta_B,\eta_U,t) -$
$2\Delta\Phi_B\Phi_U\overline{V}^{11}\mu^2\frac{\partial^2}{\partial\eta_B\partial\eta_U}P(\eta_A,\eta_B,\eta_U,t) +$
$\Delta\Phi_B\Phi_U\overline{V}^{11}\mu^2\frac{\partial^2}{\partial\eta_U^2}P(\eta_A,\eta_B,\eta_U,t) + \Delta\Phi_B\overline{V}^{\frac{21}{2}}\frac{\partial^3}{\partial\eta_B^2\partial\eta_U}P(\eta_A,\eta_B,\eta_U,t) -$
$\Delta\Phi_B\overline{V}^{\frac{21}{2}}\frac{\partial^3}{\partial\eta_B\partial\eta_U^2}P(\eta_A,\eta_B,\eta_U,t) - 2\Delta\Phi_B\overline{V}^{11}\eta_U\mu^2\frac{\partial}{\partial\eta_B}P(\eta_A,\eta_B,\eta_U,t) +$
$2\Delta\Phi_B\overline{V}^{11}\eta_U\mu^2\frac{\partial}{\partial\eta_U}P(\eta_A,\eta_B,\eta_U,t) + 2\Delta\Phi_B\overline{V}^{11}\mu^2 P(\eta_A,\eta_B,\eta_U,t) -$
$\Delta\Phi_B\overline{V}^{11}\frac{\partial^2}{\partial\eta_B^2}P(\eta_A,\eta_B,\eta_U,t) + 2\Delta\Phi_B\overline{V}^{11}\frac{\partial^2}{\partial\eta_B\partial\eta_U}P(\eta_A,\eta_B,\eta_U,t) -$
$\Delta\Phi_B\overline{V}^{11}\frac{\partial^2}{\partial\eta_U^2}P(\eta_A,\eta_B,\eta_U,t) - \frac{\Delta\Phi_B}{2}\overline{V}^{10}\frac{\partial^4}{\partial\eta_B^2\partial\eta_U^2}P(\eta_A,\eta_B,\eta_U,t) +$
$2\Delta\Phi_U\overline{V}^{11}\eta_A\mu^2\frac{\partial}{\partial\eta_A}P(\eta_A,\eta_B,\eta_U,t) - 2\Delta\Phi_U\overline{V}^{11}\eta_A\mu^2\frac{\partial}{\partial\eta_U}P(\eta_A,\eta_B,\eta_U,t) -$
$2\Delta\Phi_U\overline{V}^{11}\eta_B\mu^2\frac{\partial}{\partial\eta_B}P(\eta_A,\eta_B,\eta_U,t) + 2\Delta\Phi_U\overline{V}^{11}\eta_B\mu^2\frac{\partial}{\partial\eta_U}P(\eta_A,\eta_B,\eta_U,t) -$
$\Delta\Phi_U\overline{V}^{11}\mu^2\frac{\partial^2}{\partial\eta_A^2}P(\eta_A,\eta_B,\eta_U,t) + 2\Delta\Phi_U\overline{V}^{11}\mu^2\frac{\partial^2}{\partial\eta_A\partial\eta_U}P(\eta_A,\eta_B,\eta_U,t) +$
$\Delta\Phi_U\overline{V}^{11}\mu^2\frac{\partial^2}{\partial\eta_B^2}P(\eta_A,\eta_B,\eta_U,t) - 2\Delta\Phi_U\overline{V}^{11}\mu^2\frac{\partial^2}{\partial\eta_B\partial\eta_U}P(\eta_A,\eta_B,\eta_U,t) -$
$2\Delta\overline{V}^{\frac{21}{2}}\eta_A\frac{\partial^2}{\partial\eta_A\partial\eta_U}P(\eta_A,\eta_B,\eta_U,t) + 2\Delta\overline{V}^{\frac{21}{2}}\eta_B\frac{\partial^2}{\partial\eta_B\partial\eta_U}P(\eta_A,\eta_B,\eta_U,t) +$
$\frac{\Delta\eta_A}{2}\overline{V}^{\frac{19}{2}}\frac{\partial^4}{\partial\eta_A^2\partial\eta_U^2}P(\eta_A,\eta_B,\eta_U,t) - \frac{\Delta\eta_B}{2}\overline{V}^{\frac{19}{2}}\frac{\partial^4}{\partial\eta_B^2\partial\eta_U^2}P(\eta_A,\eta_B,\eta_U,t) +$
$\Delta\overline{V}^{\frac{19}{2}}\frac{\partial^3}{\partial\eta_A\partial\eta_U^2}P(\eta_A,\eta_B,\eta_U,t) - \Delta\overline{V}^{\frac{19}{2}}\frac{\partial^3}{\partial\eta_B\partial\eta_U^2}P(\eta_A,\eta_B,\eta_U,t) +$
$2\Delta\overline{V}^{11}\eta_A\frac{\partial}{\partial\eta_A}P(\eta_A,\eta_B,\eta_U,t) - 2\Delta\overline{V}^{11}\eta_A\frac{\partial}{\partial\eta_U}P(\eta_A,\eta_B,\eta_U,t) -$
$2\Delta\overline{V}^{11}\eta_B\frac{\partial}{\partial\eta_B}P(\eta_A,\eta_B,\eta_U,t) + 2\Delta\overline{V}^{11}\eta_B\frac{\partial}{\partial\eta_U}P(\eta_A,\eta_B,\eta_U,t) +$

$$2\Delta\overline{V}^{11}\eta_U\mu^2\frac{\partial}{\partial\eta_A}P(\eta_A,\eta_B,\eta_U,t) - 2\Delta\overline{V}^{11}\eta_U\mu^2\frac{\partial}{\partial\eta_B}P(\eta_A,\eta_B,\eta_U,t) -$$
$$\Delta\overline{V}^{10}\eta_A\frac{\partial^3}{\partial\eta_A^2\partial\eta_U}P(\eta_A,\eta_B,\eta_U,t) + \Delta\overline{V}^{10}\eta_A\frac{\partial^3}{\partial\eta_A\partial\eta_U^2}P(\eta_A,\eta_B,\eta_U,t) +$$
$$\Delta\overline{V}^{10}\eta_B\frac{\partial^3}{\partial\eta_B^2\partial\eta_U}P(\eta_A,\eta_B,\eta_U,t) - \Delta\overline{V}^{10}\eta_B\frac{\partial^3}{\partial\eta_B\partial\eta_U^2}P(\eta_A,\eta_B,\eta_U,t) -$$
$$2\Delta\overline{V}^{10}\frac{\partial^2}{\partial\eta_A\partial\eta_U}P(\eta_A,\eta_B,\eta_U,t) + 2\Delta\overline{V}^{10}\frac{\partial^2}{\partial\eta_B\partial\eta_U}P(\eta_A,\eta_B,\eta_U,t) -$$
$$2\Phi_A\Phi_B\overline{V}^{11}\mu^2 s\frac{\partial^2}{\partial\eta_A^2}P(\eta_A,\eta_B,\eta_U,t) + 4\Phi_A\Phi_B\overline{V}^{11}\mu^2 s\frac{\partial^2}{\partial\eta_A\partial\eta_U}P(\eta_A,\eta_B,\eta_U,t) -$$
$$2\Phi_A\Phi_B\overline{V}^{11}\mu^2 s\frac{\partial^2}{\partial\eta_B^2}P(\eta_A,\eta_B,\eta_U,t) + 4\Phi_A\Phi_B\overline{V}^{11}\mu^2 s\frac{\partial^2}{\partial\eta_B\partial\eta_U}P(\eta_A,\eta_B,\eta_U,t) -$$
$$4\Phi_A\Phi_B\overline{V}^{11}\mu^2 s\frac{\partial^2}{\partial\eta_U^2}P(\eta_A,\eta_B,\eta_U,t) - 2\Phi_A\Phi_U\overline{V}^{11}\mu^3\frac{\partial^2}{\partial\eta_A^2}P(\eta_A,\eta_B,\eta_U,t) +$$
$$4\Phi_A\Phi_U\overline{V}^{11}\mu^3\frac{\partial^2}{\partial\eta_A\partial\eta_U}P(\eta_A,\eta_B,\eta_U,t) - 2\Phi_A\Phi_U\overline{V}^{11}\mu^3\frac{\partial^2}{\partial\eta_U^2}P(\eta_A,\eta_B,\eta_U,t) +$$
$$2\Phi_A\overline{V}^{\frac{21}{2}}\mu\frac{\partial^3}{\partial\eta_A^2\partial\eta_U}P(\eta_A,\eta_B,\eta_U,t) - 2\Phi_A\overline{V}^{\frac{21}{2}}\mu\frac{\partial^3}{\partial\eta_A\partial\eta_U^2}P(\eta_A,\eta_B,\eta_U,t) -$$
$$4\Phi_A\overline{V}^{11}\eta_B\mu^2 s\frac{\partial}{\partial\eta_A}P(\eta_A,\eta_B,\eta_U,t) - 4\Phi_A\overline{V}^{11}\eta_B\mu^2 s\frac{\partial}{\partial\eta_B}P(\eta_A,\eta_B,\eta_U,t) +$$
$$8\Phi_A\overline{V}^{11}\eta_B\mu^2 s\frac{\partial}{\partial\eta_U}P(\eta_A,\eta_B,\eta_U,t) + 4\Phi_A\overline{V}^{11}\eta_U\mu^3\frac{\partial}{\partial\eta_A}P(\eta_A,\eta_B,\eta_U,t) -$$
$$4\Phi_A\overline{V}^{11}\eta_U\mu^3\frac{\partial}{\partial\eta_U}P(\eta_A,\eta_B,\eta_U,t) - 4\Phi_A\overline{V}^{11}\mu^3 P(\eta_A,\eta_B,\eta_U,t) -$$
$$4\Phi_A\overline{V}^{11}\mu^2 sP(\eta_A,\eta_B,\eta_U,t) - 2\Phi_A\overline{V}^{11}\mu\frac{\partial^2}{\partial\eta_A^2}P(\eta_A,\eta_B,\eta_U,t) +$$
$$4\Phi_A\overline{V}^{11}\mu\frac{\partial^2}{\partial\eta_A\partial\eta_U}P(\eta_A,\eta_B,\eta_U,t) - 2\Phi_A\overline{V}^{11}\mu\frac{\partial^2}{\partial\eta_U^2}P(\eta_A,\eta_B,\eta_U,t) -$$
$$\Phi_A\overline{V}^{10}\mu\frac{\partial^4}{\partial\eta_A^2\partial\eta_U^2}P(\eta_A,\eta_B,\eta_U,t) - 2\Phi_B\Phi_U\overline{V}^{11}\mu^3\frac{\partial^2}{\partial\eta_B^2}P(\eta_A,\eta_B,\eta_U,t) +$$
$$4\Phi_B\Phi_U\overline{V}^{11}\mu^3\frac{\partial^2}{\partial\eta_B\partial\eta_U}P(\eta_A,\eta_B,\eta_U,t) - 2\Phi_B\Phi_U\overline{V}^{11}\mu^3\frac{\partial^2}{\partial\eta_U^2}P(\eta_A,\eta_B,\eta_U,t) +$$
$$2\Phi_B\overline{V}^{\frac{21}{2}}\mu\frac{\partial^3}{\partial\eta_B^2\partial\eta_U}P(\eta_A,\eta_B,\eta_U,t) - 2\Phi_B\overline{V}^{\frac{21}{2}}\mu\frac{\partial^3}{\partial\eta_B\partial\eta_U^2}P(\eta_A,\eta_B,\eta_U,t) -$$
$$4\Phi_B\overline{V}^{11}\eta_A\mu^2 s\frac{\partial}{\partial\eta_A}P(\eta_A,\eta_B,\eta_U,t) - 4\Phi_B\overline{V}^{11}\eta_A\mu^2 s\frac{\partial}{\partial\eta_B}P(\eta_A,\eta_B,\eta_U,t) +$$
$$8\Phi_B\overline{V}^{11}\eta_A\mu^2 s\frac{\partial}{\partial\eta_U}P(\eta_A,\eta_B,\eta_U,t) + 4\Phi_B\overline{V}^{11}\eta_U\mu^3\frac{\partial}{\partial\eta_B}P(\eta_A,\eta_B,\eta_U,t) -$$
$$4\Phi_B\overline{V}^{11}\eta_U\mu^3\frac{\partial}{\partial\eta_U}P(\eta_A,\eta_B,\eta_U,t) - 4\Phi_B\overline{V}^{11}\mu^3 P(\eta_A,\eta_B,\eta_U,t) -$$
$$4\Phi_B\overline{V}^{11}\mu^2 sP(\eta_A,\eta_B,\eta_U,t) - 2\Phi_B\overline{V}^{11}\mu\frac{\partial^2}{\partial\eta_B^2}P(\eta_A,\eta_B,\eta_U,t) +$$
$$4\Phi_B\overline{V}^{11}\mu\frac{\partial^2}{\partial\eta_B\partial\eta_U}P(\eta_A,\eta_B,\eta_U,t) - 2\Phi_B\overline{V}^{11}\mu\frac{\partial^2}{\partial\eta_U^2}P(\eta_A,\eta_B,\eta_U,t) -$$
$$\Phi_B\overline{V}^{10}\mu\frac{\partial^4}{\partial\eta_B^2\partial\eta_U^2}P(\eta_A,\eta_B,\eta_U,t) + 4\Phi_U\overline{V}^{11}\eta_A\mu^3\frac{\partial}{\partial\eta_A}P(\eta_A,\eta_B,\eta_U,t) -$$
$$4\Phi_U\overline{V}^{11}\eta_A\mu^3\frac{\partial}{\partial\eta_U}P(\eta_A,\eta_B,\eta_U,t) + 4\Phi_U\overline{V}^{11}\eta_B\mu^3\frac{\partial}{\partial\eta_B}P(\eta_A,\eta_B,\eta_U,t) -$$
$$4\Phi_U\overline{V}^{11}\eta_B\mu^3\frac{\partial}{\partial\eta_U}P(\eta_A,\eta_B,\eta_U,t) + 8\Phi_U\overline{V}^{11}\mu^3 P(\eta_A,\eta_B,\eta_U,t) -$$
$$2\Phi_U\overline{V}^{11}\mu^3\frac{\partial^2}{\partial\eta_A^2}P(\eta_A,\eta_B,\eta_U,t) + 4\Phi_U\overline{V}^{11}\mu^3\frac{\partial^2}{\partial\eta_A\partial\eta_U}P(\eta_A,\eta_B,\eta_U,t) -$$
$$2\Phi_U\overline{V}^{11}\mu^3\frac{\partial^2}{\partial\eta_B^2}P(\eta_A,\eta_B,\eta_U,t) + 4\Phi_U\overline{V}^{11}\mu^3\frac{\partial^2}{\partial\eta_B\partial\eta_U}P(\eta_A,\eta_B,\eta_U,t) -$$
$$4\Phi_U\overline{V}^{11}\mu^3\frac{\partial^2}{\partial\eta_U^2}P(\eta_A,\eta_B,\eta_U,t) + 4\overline{V}^{\frac{21}{2}}\eta_A\mu\frac{\partial^2}{\partial\eta_A\partial\eta_U}P(\eta_A,\eta_B,\eta_U,t) +$$

$$4\overline{V}^{\frac{21}{2}}\eta_B\mu\frac{\partial^2}{\partial\eta_B\partial\eta_U}P(\eta_A,\eta_B,\eta_U,t) - \overline{V}^{\frac{19}{2}}\eta_A\mu\frac{\partial^4}{\partial\eta_A^2\partial\eta_U^2}P(\eta_A,\eta_B,\eta_U,t) -$$
$$\overline{V}^{\frac{19}{2}}\eta_B\mu\frac{\partial^4}{\partial\eta_B^2\partial\eta_U^2}P(\eta_A,\eta_B,\eta_U,t) - 2\overline{V}^{\frac{19}{2}}\mu\frac{\partial^3}{\partial\eta_A\partial\eta_U^2}P(\eta_A,\eta_B,\eta_U,t) -$$
$$2\overline{V}^{\frac{19}{2}}\mu\frac{\partial^3}{\partial\eta_B\partial\eta_U^2}P(\eta_A,\eta_B,\eta_U,t) - 4\overline{V}^{11}\eta_A\mu\frac{\partial}{\partial\eta_A}P(\eta_A,\eta_B,\eta_U,t) +$$
$$4\overline{V}^{11}\eta_A\mu\frac{\partial}{\partial\eta_U}P(\eta_A,\eta_B,\eta_U,t) - 4\overline{V}^{11}\eta_B\mu\frac{\partial}{\partial\eta_B}P(\eta_A,\eta_B,\eta_U,t) +$$
$$4\overline{V}^{11}\eta_B\mu\frac{\partial}{\partial\eta_U}P(\eta_A,\eta_B,\eta_U,t) + 4\overline{V}^{11}\eta_U\mu^3\frac{\partial}{\partial\eta_A}P(\eta_A,\eta_B,\eta_U,t) +$$
$$4\overline{V}^{11}\eta_U\mu^3\frac{\partial}{\partial\eta_B}P(\eta_A,\eta_B,\eta_U,t) - 8\overline{V}^{11}\eta_U\mu^3\frac{\partial}{\partial\eta_U}P(\eta_A,\eta_B,\eta_U,t) -$$
$$8\overline{V}^{11}\mu^3P(\eta_A,\eta_B,\eta_U,t) - 8\overline{V}^{11}\mu P(\eta_A,\eta_B,\eta_U,t) +$$
$$2\overline{V}^{10}\eta_A\mu\frac{\partial^3}{\partial\eta_A^2\partial\eta_U}P(\eta_A,\eta_B,\eta_U,t) - 2\overline{V}^{10}\eta_A\mu\frac{\partial^3}{\partial\eta_A\partial\eta_U^2}P(\eta_A,\eta_B,\eta_U,t) +$$
$$2\overline{V}^{10}\eta_B\mu\frac{\partial^3}{\partial\eta_B^2\partial\eta_U}P(\eta_A,\eta_B,\eta_U,t) - 2\overline{V}^{10}\eta_B\mu\frac{\partial^3}{\partial\eta_B\partial\eta_U^2}P(\eta_A,\eta_B,\eta_U,t) +$$
$$4\overline{V}^{10}\mu\frac{\partial^2}{\partial\eta_A\partial\eta_U}P(\eta_A,\eta_B,\eta_U,t) + 4\overline{V}^{10}\mu\frac{\partial^2}{\partial\eta_B\partial\eta_U}P(\eta_A,\eta_B,\eta_U,t))$$

using substitution $U := -A - B + N$.

References

- Seeley, T.D, Visscher, P.K. Schlegel, T., Hogan, P.M., Franks, N.R. & Marshall, J.A.R. (2012) Stop signals provide cross inhibition in collective decision-making by honeybee swarms. *Science* **335**, 108-111.
